# Efficacy of Acyclovir to Suppress Herpes Simplex Virus Oropharyngeal Reactivation in Patients Who Are Mechanically Ventilated: An Ancillary Study of the Preemptive Treatment for Herpesviridae (PTH) Trial

**DOI:** 10.1001/jamanetworkopen.2021.39825

**Published:** 2021-12-20

**Authors:** Charles-Edouard Luyt, David Hajage, Sonia Burrel, Sami Hraiech, Mamadou Hassimiou Diallo, Laurent Papazian, David Boutolleau

**Affiliations:** 1 Service de Médecine Intensive Réanimation, Sorbonne Université, Hôpitaux Universitaires Pitié Salpêtrière-Charles Foix, Assistance Publique-Hôpitaux de Paris, Paris, France; 2 Institut national de la Santé et de la Recherche Médicale Unité Mixte de Recherche S 1166-Institute of Cardiometabolism and Nutrition, Paris, France; 3 Sorbonne Université, Institut national de la Santé et de la Recherche Médicale, Institut Pierre Louis d’Epidémiologie et de Santé Publique, Assistance Publique-Hôpitaux de Paris, Hôpitaux Universitaires Pitié Salpêtrière-Charles Foix, Département de Santé Publique, Centre de Pharmacoépidémiologie, Centre d'Investigation Clinique-1421, Paris, France; 4 Centre National de Référence Herpèsvirus, Département de Virologie, Sorbonne Université, Hôpitaux Universitaires Pitié Salpêtrière-Charles Foix, Assistance Publique-Hôpitaux de Paris; 5 Institut National de la Santé et de la Recherche Médicale U1136, Institut Pierre Louis d'Epidémiologie et de Santé Publique, Paris, France; 6 Médecine Intensive Réanimation, Aix-Marseille Université, Hôpital Nord, Assistance Publique-Hôpitaux de Marseille, Marseille, France

## Abstract

This analysis of a randomized clinical trial examines the efficacy of acyclovir for preventing oropharyngeal HSV reactivation among patients who were mechanically ventilated.

## Introduction

In the recently published Preemptive Treatment for Herpesviridae (PTH) randomized clinical trial (NCT02152358),^1^ preemptive use of acyclovir for patients who were mechanically ventilated with oropharyngeal herpes simplex virus (HSV) reactivation failed to demonstrate a positive impact on day-60 ventilator-free days; however, a trend toward a decreased day-60 mortality rate was found in favor of acyclovir. However, the impact of acyclovir on oropharyngeal HSV reactivation was not assessed. We performed this retrospective study to evaluate the efficacy of acyclovir to suppress HSV oropharyngeal reactivation within the PTH trial.

## Methods

This analysis of a randomized clinical trial was planned in the original protocol (available with the original publication and in Supplement 1),^1^ which was approved by an independent ethics committee (Comité de Protection des Personnes Sud Méditéranée 5). Written informed consent was obtained from patients or their legally authorized representatives. For the latter, the patient’s follow-up informed consent was obtained when possible. Conduct of the study and reports of results follow the Consolidated Standards of Reporting Trials (CONSORT) reporting guideline.

The PTH trial was performed in 16 French intensive care units among 238 adults aged older than 18 years. Participants were mechanically ventilated for at least 96 hours, were expected to remain on mechanical ventilation for 48 hours or more, and had HSV oropharyngeal reactivation. Patients were randomized to receive acyclovir 5 mg/kg or a matching placebo 3 times daily for 14 days.^1^

HSV shedding was assessed on prospectively collected oropharyngeal swabs from patients included in 2 centers (Hôpital Pitié-Salpêtrière, Paris, and Hôpital Nord, Marseille) at randomization and at days 7, 14, and 21 postrandomization. All patients with at least 1 follow-up swab were included in this study (eFigure in Supplement 2). Details regarding patient sampling and sample processing are available in eMethods in Supplement 2.

Data are expressed as median (IQR), mean (SD), or mean with 95% CI, as appropriate. Between-group comparisons used Student *t* test or Mann-Whitney *U* test for continuous variables according to variable distribution (ie, normal or not normal). For categorical variables, between-group comparisons used χ^2^ or Fischer exact tests. All analyses were computed with R software version 3.5.1 (R Project for Statistical Computing) at a 2-sided 5% level of significance. Data were analyzed from February through April 2021. Further details regarding statistical analysis are given in eMethods in Supplement 2.

## Results

Among 239 patients included in the PTH trial, 133 patients were included in the 2 centers participating in this ancillary study; among these patients, 130 individuals (87 patients in Paris and 43 patients in Marseille) had at least 1 follow-up swab and were included in the study. Patient baseline characteristics were comparable between groups; among 66 patients in the acyclovir group and 64 patients in the placebo group, there were 43 (65.2%) men and 46 (71.9%) men, respectively. The groups had a median (IQR) age of 59 (50-66) years and 59 (43-66) years, admission simplified acute physiology score II of 42 (35-54) and 43 (36-50), and admission sequential organ failure assessment score of 10 (7-13) and 9 (7-12), respectively. Median (IQR) body mass index (calculated as weight in kilograms divided by height in meters squared) differed significantly between groups: 29.5 (26.2-34.4) for the acyclovir group vs 26.8 (23.4-31.4) for the placebo group.

Proportions of patients who were deceased or extubated were not significantly different from day 1 to day 21 in the acyclovir or placebo groups. However, the cumulative incidence of patients with HSV-negative oropharyngeal swabs was significantly increased in the acyclovir group from randomization to day 21 compared with the placebo group (from 0% at randomization to 67.3% at day 21 [95% CI, 49.0%-81.0%] in the acyclovir group vs 0% at randomization to 36.8% [95% CI, 23.0%-51.0%] at day 21 in the placebo group; *P* = .008) (Table and Figure). Subdistribution hazard ratios for acyclovir vs placebo obtained from the Fine-Gray model for HSV negative swab, extubation, and death were 2.50 (95% CI, 1.42-4.39), 0.41 (95% CI, 0.17-0.96), and 0.52 (95% CI, 0.20-1.35), respectively.

**Table.** Cumulative Incidence of HSV Shedding, Extubation, and Death

Outcome and treatment group	Cumulative incidence, % (95% CI)				*P* value
	Day 1^a^	Day 7	Day 14	Day 21	
HSV-negative swab					
Acyclovir	0	0.21 (0.12-0.32)	0.46 (0.33-0.58)	0.67 (0.49-0.81)	.008
Placebo	0	0.17 (0.09-0.27)	0.24 (0.14-0.36)	0.37 (0.23-0.51)	
Extubation					
Acyclovir	0.02 (0.00-0.08)^b^	0.06 (0.02-0.14)	0.10 (0.04-0.013)	0.14 (0.07-0.25)	.11
Placebo	0	0.05 (0.01-0.13)	0.14 (0.06-0.24)	0.29 (0.17-0.43)	
Death					
Acyclovir	0	0.03 (0.01-0.10)	0.09 (0.03-0.17)	0.13 (0.06-0.24)	.29
Placebo	0	0.05 (0.01-0.13)	0.11 (0.04-0.02)	0.22 (0.11-0.35)	

Abbreviation: HSV, herpes simplex virus.

^a^ Day 1 is the day of randomization.

^b^ One patient was extubated on the day of randomization.

**Figure.**
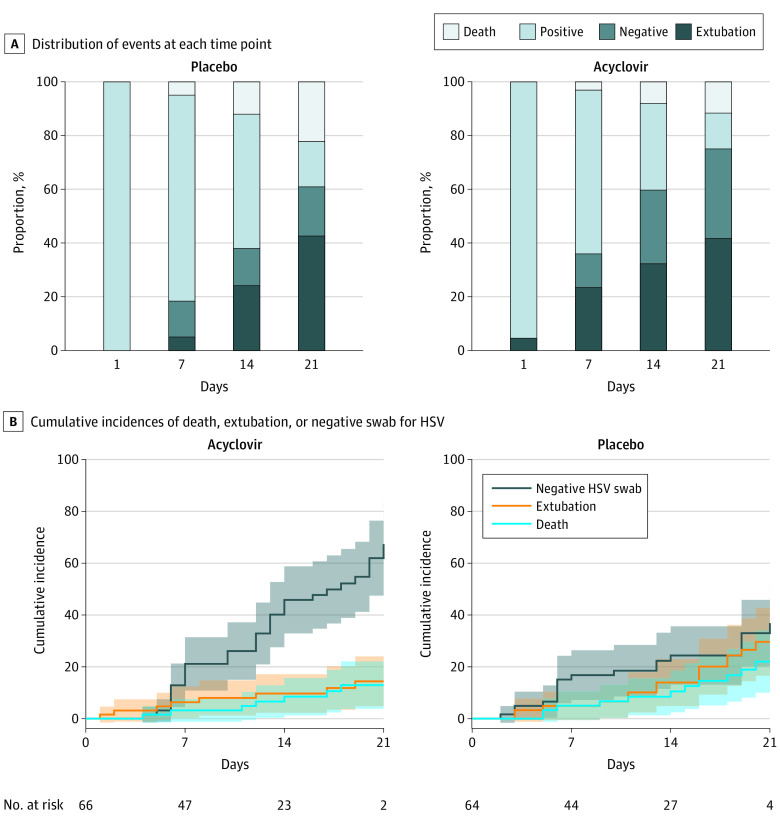
Kinetics of Herpes Simplex Virus (HSV) Shedding During First 21 Days After Randomization

## Discussion

This analysis of a randomized clinical trial found that intravenous acyclovir at a dosing of 15 mg/kg/d for 14 days was effective in decreasing the duration of HSV oropharyngeal shedding compared with placebo among patients mechanically ventilated for 96 hours or more. However, throat HSV shedding was still detected among one-third of patients after 14 days of antiviral treatment. In the parent trial,^1^ patients in the acyclovir group had a decreased mortality rate but increased duration of mechanical ventilation.

One hypothesis for the negative result of the PTH trial was the inefficacy of acyclovir to cure HSV oropharyngeal reactivation,^1^ although this drug has been shown to prevent HSV reactivation.^2^ Although we found here that acyclovir allowed the shortening of HSV shedding duration, this dosing and duration of antiviral treatment do not appear to be sufficient to totally suppress oropharyngeal HSV reactivation and shedding among patients who are mechanically ventilated. Whether increased doses and prolonged duration of treatment may lead to complete suppression of HSV shedding and may improve outcomes remains to be determined. Limitations of our study include the limited number of patients included and missing samples.

## Notes

Supplement 1.
Trial Protocol

Supplement 2.
eMethods.
**eFigure.** Study Flowchart

Supplement 3.
Data Sharing Statement

## References

[1] Luyt CE, Forel JM, Hajage D, ; Preemptive Treatment for Herpesviridae Study Group, Réseau Européen de recherche en Ventilation Artificielle Network. Acyclovir for mechanically ventilated patients with herpes simplex virus oropharyngeal reactivation: a randomized clinical trial. JAMA Intern Med. 2020;180(2):263-272. doi:10.1001/jamainternmed.2019.5713 31841577 PMC6990840

[2] Tuxen DV, Wilson JW, Cade JF. Prevention of lower respiratory herpes simplex virus infection with acyclovir in patients with the adult respiratory distress syndrome. Am Rev Respir Dis. 1987;136(2):402-405. doi:10.1164/ajrccm/136.2.402 3039882

