# Supplementary material for: Efficacy of Acyclovir to Suppress Herpes Simplex Virus Oropharyngeal Reactivation in Patients Who Are Mechanically Ventilated: An Ancillary Study of the Preemptive Treatment for Herpesviridae (PTH) Trial
Source: JAMA Netw Open. 2021 Dec 20;4(12):e2139825. doi: 10.1001/jamanetworkopen.2021.39825 (PMC8689380; doi:10.1001/jamanetworkopen.2021.39825)
Supplement: Supplement 1. — Trial Protocol [file jamanetwopen-e2139825-s001.pdf]

PTH Trial

Preemptive Treatment for *Herpesviridae*

Benefits of preemptive treatment with ganciclovir or acyclovir for patients needing prolonged mechanical ventilation and having either cytomegalovirus replication in their blood or oropharyngeal herpes simplex virus replication:
Prospective, multicenter, randomized double-blind trial

Sponsor: Assistance publique - Hôpitaux de Marseille (Marseille Public Hospitals)
Project manager: principal investigator: Pr Laurent PAPAZIAN – Intensive Care – Respiratory Distress and Severe Infections – URMITE CNRS-UMR 7278 – Aix-Marseille University, France

Intensive care research project sponsored by the European network REVA (Recherche En Ventilation Artificielle; Research on Mechanical Ventilation)

Principal Investigators:

- Pr Jean CHASTRE – Hôpital de la Pitié-Salpêtrière, APHP
- Pr Samir JABER – Hôpital Saint-Eloi, CHU Montpellier
- Dr Charles-Edouard LUYT - Hôpital de la Pitié-Salpêtrière, APHP
- Pr Laurent PAPAZIAN – Hôpital Nord, APHM
- Pr Michel WOLFF and Pr Jean-François TIMSIT- Hôpital Bichat, APHP

- **Co-Investigators:**
  - Pr Jean-Yves FAGON – HEGP, APHP
  - Pr Jean-Yves LEFRANT – Hôpital Caremeau, CHU Nîmes
  - Pr Jean-Michel CONSTANTIN – CHU Estaing, CHU Clermont-Ferrand

- **Pr Bertrand SOUWEINE – Hôpital Gabriel Montpied, CHU Clermont-Ferrand**
- **Thomas RIMMELE – Hôpital Edouard Herriot, HCL**
- **Pr Claude GUERIN – Hôpital de la Croix-Rousse, HCL**
- **Dr Jean-Pierre BEDOS – Hôpital Mignot, Versailles**
- **Pr Jean-Paul MIRA – Hôpital Cochin, APHP**
- **Dr Qin LU - Hôpital de la Pitié-Salpêtrière, APHP**
- **Pr Alexandre DUGUET - Hôpital de la Pitié-Salpêtrière, APHP**
- **Dr Marc CLAVEL – Hôpital Dupuytren, Limoges**
- **Pr Carole SCHWEBEL – Hôpital Nord, Grenoble**
- **Pr Marc LEONE – Hôpital Nord, Marseille**
- **Pr Marc GAINNIER – Hôpital de la Timone, Marseille**
- **Dr ALLARDET-SERVENT – Hôpital Européen, Marseille**
- **Dr Christine ZANDOTTI – Hôpital de la Timone, Marseille**
- **Dr Christine PENOT-RAGON – Hôpitaux Sud, Marseille**
- **Dr David BOUTOLLEAU - Hôpital de la Pitié-Salpêtrière, APHP**
  <u>Coordination and logistics</u>
- **Dr Joelle MICALLEF and Laurence ATTOLINI, CIC-CPCET, Hôpital Timone, APHM**

- **<u>Methodology and Data processing</u>**
  - **Elisabeth JOUVE, Biometrics Department, CIC-UPCET, AP-HM**
  - **Pr Xavier THIRION and Vanessa PAULY, SSPIM Sud, AP-HM**
  - **Pr Alain MALLET, URC Pitié-Salpêtrière, APHP**

Summary

**Trial objective**	For patients under mechanical ventilation for at least 96 hours, to show that ganciclovir treatment of patients with CMV replication or acyclovir treatment of those with HSV replication enables to reduce the length of mechanical ventilation and improves prognosis.
**Type of trial**	Dual interventional multicenter prospective, randomized, placebo-controlled, double-blind trial with a common patient selection process: patients are jointly screened twice a week for CMV replication in blood and oropharyngeal HSV replication, then included in one of the arms (CMV arm or HSV arm) according to the type of reactivation (CMV or HSV respectively).
**Primary evaluation criterion (identical for both study subsets)**	**Number of ventilator-free days and alive at 60 days after randomization.**
**Main secondary evaluation criteria**	- Mortality at 60d, in ICU and in hospital<br>- Total length of mechanical ventilation in survivors<br>- Total length of stay in ICU and in hospital<br>- Incidence of bacterial pneumonia acquired under mechanical ventilation and of bacteremia<br>- Sequential organ failure assessed by SOFA score<br>- Appearance of side effects linked to acyclovir (kidney failure) or to ganciclovir (myelotoxicity)<br>- Disappearance of oropharyngeal HSV reactivation and CMV PCR in blood becoming negative<br>- Onset of herpes simplex pneumonia or of active CMV infection
**Selection of patients eligible for the 2 study subsets**	Patients hospitalized in ICU, under mechanical ventilation for the past 4 days, with further mechanical ventilation planned for >48 hours and with no exclusion criteria were screened twice per week:<br>- for CMV replication in the blood by PCR until extubation, death or inclusion in the HSV arm<br>- for oropharyngeal HSV replication by PCR from a throat swab until extubation, death or inclusion in the CMV arm
**Inclusion criteria**	Patients hospitalized in ICU, under mechanical ventilation for at least the past 4 days and<br>- for the CMV arm: first show CMV replication in blood<br>- for the HSV arm: first show oropharyngeal HSV replication
**Main exclusion criteria**	Age <18 years, immunodepression (HIV, with bone marrow or solid organ graft, under immunosuppressive therapy including steroids at a dose >0.5mg/kg/day for over one month), pregnant, under acyclovir, ganciclovir or foscavir treatment within the previous 30 days, morbidity or a decision to limit active treatment, active CMV or HSV infection, severe neutropenia, severe thrombocytopenia, hypersensitivity to ganciclovir (in CMV arm) or to acyclovir (in HSV arm).
**Treatments under study**	For the CMV subset, patients randomized into the ganciclovir arm will be given 5 mg of ganciclovir/kg twice a day (10 mg/kg/d) for 14 days. In the event of kidney failure, doses shall be adapted to renal function. Patients randomized into the placebo arm will be given a placebo with an identical presentation to ganciclovir.<br>For the HSV subset, patients randomized into the acyclovir arm will be given 5 mg acyclovir/kg 3 times a day (15 mg/kg/d) for 14 days. Patients randomized into the placebo arm will be given a placebo with an identical presentation to acyclovir.<br>In the event of kidney failure, doses shall be adapted to renal function.
**Number of patients**	The hypothesis is that ganciclovir or acyclovir therapy would increase the number of days alive without mechanical ventilation by 8 days calculated

	over 60 days compared to a placebo. Accordingly, 112 patients must be included in each arm. In order to ensure obtaining 112 patients per arm, we plan to include 240 patients in total for each arm, i.e. a total of 480 patients.

Table of contents

TABLE OF CONTENTS 5
1. INTRODUCTION AND JUSTIFICATION FOR RESEARCH 8
Cytomegalovirus 8
Incidence in intensive care and methods of detection 8
Manifestations: virus does not mean "viral infection" 9
Antiviral treatment of CMV: with what and for who? 9
Herpes simplex virus type 1 10
Herpes simplex virus type 1 in intensive care 10
1. HSV : pathogen or witness of the severity of an underlying disease? 11
2. Rationale for a randomized trial evaluating acyclovir 12
A project in common to pool efforts 12
Task-sharing to optimize response time for the questions asked 13
2. RESEARCH OBJECTIVES 14
Hypothesis tested 14
Primary objective 14
Secondary objectives 14
3. EXPERIMENTAL PLAN 15
4. 4. STUDY POPULATION 17
Multicenter recruitment 17
Patient selection criteria 17
Inclusion criteria 17
Exclusion criteria 18
Choice and threshold of PCR 18
4.5.1 CMV 18
4.5.2 HSV 19
5. TREATMENTS UNDER STUDY 19
Ganciclovir 19
Acyclovir 21
Placebos 22
Packaging, labeling and storage conditions 22
3. Distribution 25
Early treatment 26
Responsibility concerning the treatment under study 26
Criteria for stopping the treatment under study 27
4. Unblinding 27
Compliance 27
Treatment returns and destruction 28
6. EVALUATION CRITERIA 28
Main judgment criterion: number of days alive and weaned off MV defined at D60 28
Secondary judgment criteria 29
5. Evaluation of tolerance 30
6. Other parameters to be noted 30
7. STUDY COURSE 31
Selection 31
Inclusion 31
Randomization 31
Monitoring from D1 to D14 after inclusion 31
Monitoring from D14 to D60 32
Virology monitoring from D1 to D30 32
Visit on D60 32

Summary of patient monitoring ... 32
Treatment of declared HSV or CMV infection ... 32
Early withdrawal from the trial ... 33

8. DATA COLLECTION AND MONITORING ... 33

Case report form ... 33
Role of CRA coordinators ... 34
Quality control ... 34

9. DATA MANAGEMENT ... 34

Data management during the trial ... 34
Data coding ... 35
Data validation ... 35
Review of data validation ... 35
Freezing the database ... 35
Data storage ... 35

10. STATISTICAL METHOD ... 35

Planned number of patients and justification ... 35
Randomization method ... 36
Statistical analysis ... 37
Withdrawal from the trial and deviations from the protocol ... 37
Populations analyzed ... 37
General statistical approach ... 38
Description of patients and comparability between groups ... 39
Efficiency analysis ... 39

11. TREATMENTS ... 41

7. Treatment under study ... 41
8. Concomitant treatment ... 41

12. STUDY CALENDAR ... 42

Study length ... 42
Length of participation of each person ... 42
Research calendar ... 42

13. ADVERSE EVENT MANAGEMENT ... 42

9. General Definition ... 42
10. Definition of an expected serious adverse event ... 43
11. Definition of an unexpected serious adverse event ... 43
12. Intensity criteria ... 43
13. Conduct to be followed in the case of a serious adverse event ... 44
14. SAE follow-up ... 45

14. SURVEILLANCE COMMITTEE ... 45

Role ... 45
Composition ... 45

15. LEGAL AND ETHICAL ASPECTS ... 46

Sponsor's role ... 46
Quality control and quality assurance ... 47
Submission to the ethics committee ... 47
Data processing – Confidentiality ... 47
Criteria for stopping the trial early ... 48

16. PUBLICATION RULES ... 49

17. COST AND ADDITIONAL COSTS OF RESEARCH ... 49

18. RESPONSIBILITIES OF THE SPONSOR / INSURANCE ... 49

19. REFERENCES ... 50

20. ANNEXES 52
15. LIST OF THE CENTERS HAVING ACCEPTED TO PARTICIPATE 52
16. CLINICAL PULMONARY INFECTION SCORE (CPIS) 53

1. Introduction and justification for research

Viral infections have only very recently become an object of investigation in patients under mechanical ventilation (MV) hospitalized in intensive care unit (ICU) (1-4). It is mainly the progress that has been made in directly detecting these pathogens that has enabled intensive care specialists to evaluate the impact of viruses on their patients more objectively. This of course includes "community" viral infections (influenza, rhinovirus…), but also viral infections in immunocompetent adults under MV. These are known as "nosocomial" viral infections among which *Herpesviridae* are the most frequently encountered and the most studied (1-3). *Herpesviridae* replication in intensive care patients is more often reactivation than a primary infection. This reactivation results from the fact that after a few days of MV there is an onset of immunoparalysis which can pave the way for bacterial or viral infections (5).

Cytomegalovirus

Cytomegalovirus (CMV) is a DNA virus belonging to the *Herpesviridae* family beginning with a primary infection during childhood which is usually totally or partially asymptomatic (6). Afterwards, the virus remains latent in cells of the immune system, however a drop in the host's cellular immunity (transplantation, AIDS, immunosuppressive therapy…) can cause an endogenous recurrence of viral infection.

CMV seroprevalence in adults is higher than 60% in industrialized countries (6), and from our experience, 75% on hospital admission (1). Patients in intensive care essentially have a reactivation of endogenous CMV (10), however infection by exogenous CMV remains possible (ex: transfusion) though rare, especially since systematic leukodepletion of blood products in France.

Incidence in intensive care and methods of detection

The incidence of active CMV infection in ICU patients is between 15 and 20% (1, 3, 7-11). Patients hospitalized for severe septicemia are probably at a higher risk of subsequent CMV reactivation (8, 12), as are patients who are CMV seropositive on hospital admission (1). Incidence variability also depends on the method chosen to screen CMV. Indeed, some methods such as PCR or pp65 antigenemia of the blood are more sensitive than the culture of respiratory secretions and/or the presence of a cytopathic effect in bronchoalveolar lavage (BAL).

Finally, considering all of the patients admitted to intensive care unit (ICU) and monitored by weekly antigenemia assays, CMV infection concerns at least 1 out of 6 patients (1).

Manifestations: virus does not mean "viral infection"

On the contrary to immunodepressed patients (6), extra-pulmonary clinical manifestations (colitis, retinitis) are very rarely reported in ICU patients (13). It was a study on lung histology that revealed the reality of CMV infection in immunocompetent patients, this virus could be considered as a pathogen responsible for ventilator-associated pneumonia (VAP) (12). In a study on surgical lung biopsies from 86 patients with unexplained respiratory distress, 25 had histological signs of CMV lung infection. The same group then identified the same signs in 30 out of 100 patients who had had a lung biopsy for persistent ARDS (14). Furthermore, patients with CMV infection had a much higher rate of morbidity (length of MV and length of ICU stay) and/ or of mortality than other patients, independently of other prognostic factors (3). Finally, some experimental arguments also suggest that CMV has a genuine pathogenicity. Thus, in mice pre-infected with CMV, sepsis induced CMV reactivation in the lungs (7).

Nevertheless, CMV reactivation detected by PCR or antigenemia in intensive care patients is not necessarily synonymous with CMV disease. Indeed a certain number of these reactivations clear without any antiviral treatment, suggesting that CMV may only be a marker of the degree of immunodepression acquired in intensive care. **Only a therapeutic trial testing the impact of an antiviral drug could provide undeniable evidence of the pathogenicity of CMV for these patients.**

Antiviral treatment of CMV: with what and for whom?

Antiviral drugs have adverse effects preventing an empirical proposal of systematic prophylaxis on admission, whereas only 1/3 patients at the most are concerned. Apart from the cases where a lung biopsy is performed (curative treatment), ICU specialists sometimes start a "preemptive" treatment as soon as early reactivation markers become positive (antigenemia or PCR), and even more so in cases where there is no other explanation for correctly investigated respiratory distress and/or when steroid therapy becomes necessary. In these cases ganciclovir is used. Up until now there have been no studies on the pertinence of a preemptive strategy with regards to CMV. Only a large-scale, interventional multicenter trial could evaluate the pertinence of such a **therapeutic strategy** (efficiency, tolerance).

Herpes simplex virus type 1

Herpes simplex virus (HSV)-1 is a DNA virus belonging to the *Herpesviridae* family. Like CMV, it infects humans during their early childhood: primary infection can be asymptomatic (the majority of cases) or symptomatic, in which case it appears as herpetic gingivostomatitis. The virus remains latent in the sensory ganglia of the spinal cord (the most often in trigeminal ganglia), and in the event of trauma, endocrine stimulation or immunodepression, it can be reactivated and either induces an asymptomatic saliva excretion, or lip sores (“cold sores”). By the age of 50, 90% of adults are thought to be HSV seropositive.

Herpes simplex virus type 1 in ICU

HSV-1 was isolated in the saliva of 1–5 % of the general population. In ICU, the rate of viral reactivation is higher. A recent study showed that 22% of patients hospitalized in ICU expressed HSV-1 in the throat (15), while 41% of patients expressed it after surgery (16). In a study on 201 immunocompetent patients who had been on MV for at least 5 days, HSV was detected in the throat of 109 (54%) patients. Viral reactivation was asymptomatic in 56% of these cases, whereas it was expressed as herpetic lip sores or gingivostomatitis in 48 (44%) of the 109 patients with viral reactivation(2).

HSV was detected in the distal airways of 16 to 64% of patients hospitalized in ICU, this variation depended on the case mix and the method of diagnosis used. For patients hospitalized in ICU and with unselected MV (both short- and long-term ventilation), the frequency is between 15 and 25%: Bruynseels *et al.* found HSV in 16% of mechanically ventilated patients (15), Ong *et al.* detected HSV in 27% of their ventilated patients (17). When the patients have been on MV for at least 4 days, the incidence of HSV reactivation in distal airways is even higher, at over 50%: Luyt et al., in a prospective series of 201 immunocompetent patients ventilated for over 5 days, found HSV by PCR or viral culture of BAL in 64% of patients (2). De Vos et al., in a population ventilated for at least 48h, found a 62% incidence (18).

However, the detection of HSV in the lower respiratory tract does not mean that there is viral damage of the lung parenchyma, HSV detection may result from contamination of the airways with saliva, from tracheobronchial reactivation without parenchyma damage, or from genuine HSV pneumonia. This HSV pneumonia was first described in immunodepressed patients (19), with acute respiratory distress syndrome (ARDS) (20), after surgery (21) or in

burns patients (22). Luyt et al. recently showed that HSV bronchopneumonitis was common in immunocompetent patients with long periods of MV; in a study assessing 201 patients suspected of ventilation-associated pneumonia, 21% suffered from histologically proved HSV bronchopneumonitis (defined by the association of clinical worsening, the presence of virus in the tracheobronchial tree and the presence of specific nuclear inclusions in cells collected from BAL) (2).

1. HSV : pathogen or witness of the severity of an underlying disease?

There are data in the literature in favor of a genuine pathogenicity of HSV in immunocompetent patients hospitalized in ICU. In 1982, Tuxen *et al.* found that 30% of patients with ARDS who had a lung biopsy had histological proof of herpes damage (20). These patients had longer lengths of MV and hospitalization than patients with ARDS without histologically proven herpes damage (20). In the study by Bruynseels *et al.*, patients with HSV detected in the throat had a higher rate of mortality and a longer length of MV than those with no HSV detected. However, this association was not found in multivariate analysis (15). In the study by Luyt *et al.* on patients with at least 5 days of MV, the patients who developed HSV bronchopneumonitis had longer lengths of MV and hospitalization in intensive care than paired control patients without HSV pneumonia. Furthermore, patients with HSV bronchopneumonitis could develop nosocomial bacterial pneumonia more frequently than others, but with an identical rate of mortality (2).

More recently, Linssen *et al.* found that patients with an HSV viral load above 10^5 copies/ml in BAL had an adjusted mortality 14 days higher than those with a viral load $<10^5$ copies/ml (23). Yet the viral load in BAL was shown to be associated with the development of HSV pulmonary damage (2).

If the replication of HSV in the lower respiratory tract (and even more so in cases of genuine HSV bronchopneumonitis) is associated with increased morbidity and maybe mortality, this is only an association and there will still be a controversy about the actual pathogenicity of HSV in ventilated patients: is it a genuine parenchymal disease with its own morbidity/mortality (autonomous pulmonary damage paving the way for bacterial secondary infections), or is it just a marker for the severity of an underlying disease? There are no data in literature to currently answer this question. The only randomized study was performed by Tuxen in 1987, who randomly allocated 45 patients with ARDS for the preventive administration of acyclovir or a placebo. The results of this study showed that preventive

therapy enabled to reduce the incidence of HSV reactivation but had no effect on the length of ventilation or on mortality (24). However, this study was small and it was not possible to formally conclude that the treatment had no efficacy for these patients.

2. Rationale for a randomized trial evaluating acyclovir

The fact that HSV reactivation is potentially pathogenic for ICU patients (via HSV pneumonia, itself responsible for morbi-mortality) and the lack of randomized studies in scientific literature assessing the efficacy of preemptive acyclovir treatment for patients, justifies carrying out a randomized study in order to answer the question of whether preemptive treatment of these patients is useful.

The choice of the population studied, i.e. patients with oropharyngeal HSV reactivation is linked to the pathophysiology of lung damage: first a primary reactivation of HSV in the oropharynx, then a secondary infection of subglottic airways via micro-inhalations. In the majority of cases, there is viral reactivation in the distal airways but in certain cases this replication leads to actual histological lung damage (pneumonia). Treating patients at the beginning of the cycle, during oropharyngeal replication of HSV would prevent the contamination of distal airways and thus prevent HSV pneumonia.

The choice of preemptive rather than curative treatment is justified by the fact that when lung damage is declared, it is probably too late and treatment is probably less beneficial: by analogy, acyclovir or valacyclovir treatment is less efficient on herpetic lip sores once the sores are visible. Furthermore, HSV bronchopneumonitis is difficult to diagnose in ICU patients, requiring histological confirmation or quantification of the viral load, 2 exams which are difficult to implement in daily practice, and even more so as screening for a multicenter trial (2). On the contrary, preventive (prophylactic) treatment of all intubated patients under MV for longer than 4 days with acyclovir could be discussed; however, this would expose patients without HSV reactivation to a potentially toxic treatment.

A project in common to pool efforts

There is a strong rationale to submit a project in common including both study subsets on *Herpesviridae*

- The same issue for both *Herpesviridae*: does the replication of one of the 2 viruses in intensive care have its own morbi-mortality? Does it justify for specific preemptive treatment?

- An identical target population for both *Herpesviridae*: patients who have been on MV for at least 4 days probably justifying for prolonged MV
- Coupling the screening for both *Herpesviridae*, makes it possible to optimize implementation, reduce the work load of intensive care teams and cut costs
- An identical study design for both study subsets after patient randomization. Performing 2 study subsets in parallel in the same centers will again enable to optimize the means used, cut costs, and above all, to be sure to benefit from the expertise of investigation centers in the field of clinical trial implementation and especially that of MV and its complications
- Conducting 2 study subsets in parallel makes it possible to obtain supplementary data on the epidemiology of both viruses; especially whether the replication of CMV is associated to that of HSV, and vice versa? Is ganciclovir treatment, potentially active against HSV, able to prevent HSV parenchymal lung damage? And is acyclovir, efficient at high doses in preventing CMV reactivation in organ transplant patients, able to prevent CMV replication?
- Finally, a collaborative project like this would create a synergy of expertise acquired in both of these domains by the principal investigators (1, 2, 9, 11)

Task-sharing to optimize response time for the questions asked

Each respective arm will be managed by the following separate teams

- the CMV arm by Pr Papazian and Pr Jaber's teams (Marseille and Montpellier)
- the HSV arm by Pr Chastre and Pr Wolff's teams (Paris).

2. Research objectives

Hypothesis tested

Our hypothesis is that the administration of an **anti-viral** drug (ganciclovir for CMV, acyclovir for HSV) **as soon as PCR results are positive** (blood for CMV, oropharyngeal for HSV) **enables to increase the number of ventilator-free days and alive** (VFD) after 60 days (the number of days for which the patient is alive and weaned off MV) (d1 = the day ganciclovir, acyclovir or a placebo treatment is started) for patients treated with ganciclovir compared to the placebo on the one hand (CMV arm), and for patients treated with acyclovir compared to the placebo on the other hand (HSV arm).

Primary objective

An increase by at least 8 days of the **number of days the patients are weaned off MVand alive** at 60d (VFD D60) post-inclusion:

- with a 14d treatment of ganciclovir following positive CMV PCR results in blood
- with a 14d treatment of acyclovir following positive oropharyngeal HSV PCR results.

The number of ventilator-free days is calculated after the absence of any invasive MV for at least 48h.

Secondary objectives

The following will be studied for each arm:

- Mortality at D60
- ICU mortality
- Hospital mortality
- Length of invasive MV
- Length of ICU stay
- Length of hospital stay
- Incidence of active CMV infections – Rates of reactivation
- Incidence of HSV bronchopneumonitis
- Sequential organ failure on D3, D5, D7, D14, D21 and D28 assessed using the SOFA score
- Incidence of bacterial infections (ventilator-associated pneumonia – bacteremia)

- Incidence of ARDS
- Incidence of septic shock
- CMV PCR results becoming negative
- Disappearance of oropharyngeal HSV reactivation
- Tolerance to treatments under study: especially adverse effects of ganciclovir (myelotoxicity) and of acyclovir (kidney failure)
- Virology ancillary studies
    - Incidence and diagnostic value of HSV viremia (same tube as the one taken for CMV) for patients included in the interventional study (no extra sample taken)
    - Genetic variability of HSV strains isolated from the oropharyngeal sample leading to the inclusion of patients in the interventional study (no extra sample taken)
    - Detection of community respiratory viruses by multiplex PCR of the oropharyngeal sample leading to the inclusion of patients in the interventional study (no extra sample taken).

3. Experimental plan

It is a **prospective trial split into two arms, randomized, comparative and double-blind carried out on 2 parallel groups of 2.**

- CMV arm: one experimental group using ganciclovir and one group with standard care and a placebo in case of positive CMV PCR results in blood.
- HSV arm: one experimental group using acyclovir and one group with standard care and a placebo in case of positive oropharyngeal HSV PCR results.

Screening: Intensive care patients ventilated for over four days are monitored using PCR (quantitative in blood for CMV, qualitative from oropharyngeal swab for HSV) twice a week. Their CMV serologic status is determined using the first PCR results. This CMV and HSV monitoring is performed for as long as the patients are under MV in ICU and for a maximum of 30 days of MV. Screening ends after 30 full days under MV, or leaving intensive care or death. Once the patient is included in the trial, the systematic PCR results are no longer transmitted to the clinicians (unless the clinician specifically asks for them when there is suspected active infection). **It should be noted that all of the centers collect all of these samples routinely. This screening does not therefore modify usual patient care.**

The early start of antiviral treatment requires the inclusion of patients as soon as PCR results are positive (blood for CMV or oropharynx for HSV). The patient is included in only one arm of the trial. In the CMV arm if the first positive PCR results are CMV PCR. The HSV arm if the first positive PCR results are HSV PCR. In the case of simultaneous positive PCR results the patient is included in the CMV arm. The treatment (ganciclovir or placebo for CMV / acyclovir or placebo for HSV) is stopped when the patient leaves the ICU if the patient is completely weaned off invasive MV (intubation or tracheotomy) or at the latest 14 days post-inclusion.

Study diagram

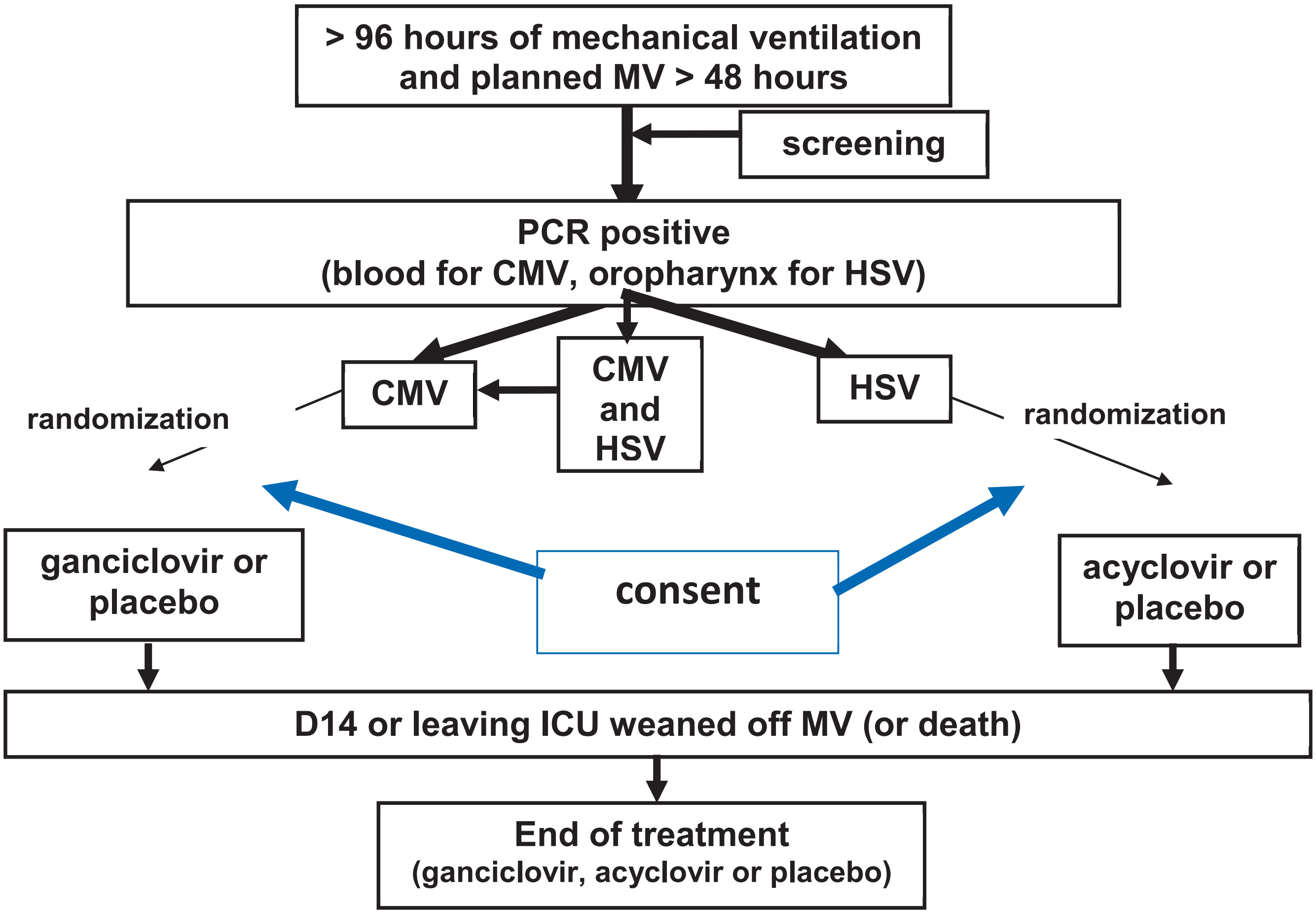

4. 4. Study population

Multicenter recruitment

Given the number of patients to be included and the units' capacity of recruitment, the centers participating in this trial should make it possible to answer the questions being asked. Each coordinator will have to classify the patients susceptible of being included, and if appropriate, the reasons why they are excluded. All of the screened patients will therefore be classified, regardless of virology results. The list of centers is displayed in Annex 1. We count on 15 inclusions per center and per year, i.e. approximately one inclusion every three weeks.

Patient selection criteria

Selection of patients eligible for both arms of the study: All patients hospitalized in ICU, under MV for the past 4 days, with ventilation planned to last at least 48 hours longer and without any exclusion criteria will be screened twice a week:

- for CMV replication by PCR on blood until 30 full days under MV, leaving ICU or death.
- For oropharyngeal HSV replication by PCR on a throat swab until 30 full days under MV, leaving ICU or death.

Inclusion criteria

Patients with all of the following criteria will be eligible for inclusion in either arm of the study depending on the type of positive PCR results:

- invasive MV for at least the past 96 hours and planned to last for at least 48 hours longer
- PCR positive (see below for PCR positivity criteria)
- aged at least 18
- with written consent from the patient, from a close relative or from the person of trust previously appointed.
- under social security cover
- negative pregnancy test for women of childbearing age

Exclusion criteria

Patients with at least one of the following criteria will not be eligible:

- Patients aged under 18

- Patients deprived of freedom or under legal protection
- Patients not covered by social security
- Patient taking ganciclovir or acyclovir
- Other antiviral drugs active against CMV and/or HSV taken orally in the month prior to inclusion (cidofovir, foscarnet, ganciclovir, or valacyclovir)
- Infection by CMV or by HSV treated by oral route in the month prior to admission in intensive care
- Hypersensitivity to ganciclovir for the CMV arm or to acyclovir for the HSV arm
- Pregnancy
- Breast-feeding
- Patients hospitalized for bone marrow aplasia
- Solid organ or bone marrow transplant patients
- Patients who are HIV-carriers with known diagnosis
- Immunosuppressive treatment (including steroids at a dose > 0.5mg/kg/day of prednisone or equivalent for more than the past month)
- Patients who are dying defined by a SAPS II score calculated over the first 6 hours prior to inclusion > 75 points
- Patients for whom it has been decided to limit active treatment
- Neutropenia ≤ $500/mm^3$ for the CMV arm
- Thrombocytopenia < $25000/mm^3$ for the CMV arm
- Patients readmitted into intensive care during the same hospital stay: the second stay does not count if they were screened during the first stay

Choice and threshold of PCR

4.5.1 CMV

- **Sample:** EDTA whole blood
- **Viral quantification: quantitative CMV PCR** following extraction and amplification conditions set up in the local virology laboratory for diagnostic activities (techniques developed and validated by the laboratory or an EC labeled kit).
- **Presentation of results:** international units (IU) /mL of whole blood (an international WHO standard for CMV exists). The threshold of therapeutic intervention is set at 500 IU/mL whole blood. The threshold thus expressed in IU will be applied in all of the centers participating in the PTH protocol. Furthermore quality control will be

organized by the Marseille laboratory in order to check that each of the laboratories quantifies in a similar fashion a sample with a CMV viral load close to this threshold of therapeutic intervention.

- **Quality assurance:** each of the laboratories participates in European quality control for CMV QCMD (*Quality Control for Molecular Diagnostics*)

4.5.2 HSV

- **Sample:** systematic throat swab (or swab of a mouth sore suspected of being herpetic) using Sigma Virocult® (MW951S).
- **Viral detection: qualitative HSV PCR** according to extraction and amplification conditions set up in the local virology laboratory for diagnostic activities (techniques developed and validated by the laboratory or an EC labeled kit).
- **Presentation of results:** positive or negative
- **Quality assurance:** each of the laboratories participates in European quality control for HSV QCMD (*Quality Control for Molecular Diagnostics*).

5. Treatments under study

Ganciclovir

Rationale: ganciclovir is the most active antiviral drug against CMV available as an injectable solution along with Foscarnet. Renal toxicity of the latter limits its use. Since all of the patients included are under MV, it is essential to use a drug which can be injected.

Description of the experimental drug: As of February 14, 2016, the product used to prepare the experimental drug for the CMV arm of the clinical trial is Cymevan™ 500 mg, a powder for intravenous use (perfusion) commercialized by Roche. The reference document for this product is the Summary of Product Characteristics (SmPC) Cymevan 500 mg, Powder for intravenous use (perfusion). Individuals who have been allocated to the CMV arm will be administered ganciclovir extemporaneously reconstituted in 5% glucose solution (50 or 100 ml). The placebo shall be made up of only 5% glucose solution (50 or 100 ml). Individuals allocated to the placebo arm will be administered a perfusion of 5% glucose solution (50 or 100 ml) in exactly the same way as ganciclovir is administered.

Dosage and methods of administration: ganciclovir is administered intravenously in one hour at a dose of 5 mg/kg, twice a day for patients with normal renal function (creatinine <

120 µmol/l). For patients with altered renal function, ganciclovir perfusions will follow the MA recommendations of 05/10/2001, which are:

- Creatinine between 120 and 220 µmol/l or clearance 25-50 ml/min/1.73m²: 2.5 mg/kg x 2 / day
- Creatinine between 221 and 400 µmol/l or clearance 10-25 ml/min/1.73m²: 2.5 mg/kg x 1 / day
- Creatinine > 400 µmol/l or clearance 0-10 ml/min/1.73m²: 1.25 mg/kg x 1 / day
- Intermittent hemodialysis: 1.25 mg/kg after dialysis session
- Continuous venovenous hemo(dia)filtration: 1.25 mg/kg x 1 / day (Bourquin et coll. Dose adaptation of drugs commonly used in intensive care during continuous renal replacement therapy (in French). Néphrologie et Thérapeutique (2009) doi : 10.1016/j.nephro.2009.02.010).

The product is prepared in compliance with the method described in the SmPC. This is carried out by the nurse. As of February 14, 2016, ganciclovir will be reconstituted and dispensed with the label "clinical trial" by the pharmacy of each site which is authorized to carry out preparations made necessary for biomedical research including experimental drugs. In the event of it being impossible for the PUI to prepare the treatment under study (too many demands for clinical study preparations, less staff on weekends and bank holidays), the latter will dispense the product labeled "clinical trial" to a third-party unit not involved in the PTH trial. The verum treatment **labeled "clinical trial"**, will be prepared according to the SmPC of the product (ganciclovir). The list of "third-party" units is provided in section 20.1 of this protocol. A specific prescription is provided for when a "third-party" unit is called for.

Length of treatment: treatment shall be administered for 14 days

<u>Concomitant treatment with Ganciclovir</u>

<u>COMBINATION ADVISED AGAINST</u>

didanosine: on the one hand, a risk of increasing didanosine side effects due to a significant increase of its serum concentration; on the other hand, a risk of reducing ganciclovir efficacy by reducing its serum concentration, if both drugs are administered within 2 hours of each other.

<u>COMBINATIONS REQUIRING USE OF PRECAUTION</u>

- **zidovudine**: increased hematologic toxicity (added effect of bone marrow toxicity). Temporarily stop taking zidovudine; monitor blood count and, if possible, reintroduce zidovudine at a low dose.

- **zalcitabine**: increased risk of peripheral neuropathy by adding adverse effects. Regular clinical monitoring.

- **imipenem-cilastatine**: occurrence of seizures of uncertain accountability.

- more generally, some prudence is recommended when concomitantly using ganciclovir and potentially hematotoxic products such as linezolide for example, which requires blood count monitoring every other day as the ANSM (French National Agency for Drug Safety) recommend for this protocol.

Acyclovir

Rationale: Acyclovir is the injectable drug of reference in HSV treatment.

Product description: The product used in the HSV arm of this clinical study is Acyclovir MYLAN® 500 mg, powder for IV injectable solution, commercialized by Mylan. The reference document for this product is the Summary of Product Characteristics of the product Acyclovir 500 mg, Powder for perfusion (IV)

Dosage and methods of administration: acyclovir is administered intravenously in one hour at a dose of 5 mg/kg 3 times per day (15 mg/kg/day) for patients with normal renal function (creatinine clearance > 50 ml/min). For patients with altered renal function, perfusions will follow the MA recommendations of 05/11/1998, which are:

- Creatinine clearance between 25 and 50 ml/min : 5 mg/kg x 2 / day
- Creatinine clearance between 10 and 25 ml/min : 5 mg/kg x 1 / day
- Creatinine clearance between 0 and 10 ml/min : 2.5 mg/kg x 1 /day
- Intermittent dialysis: 2.5 mg/kg after dialysis session
- Continuous venovenous hemo(dia)filtration: 5 mg/kg x 1 / day (Bourquin et coll. Dose adaptation of drugs commonly used in intensive care during continuous renal replacement therapy (in French). Néphrologie et Thérapeutique (2009) doi : 10.1016/j.nephro.2009.02.010).

The product is prepared in compliance with the method described in the SmPC. This is carried out by the nurse. As of November 16, 2016, acyclovir will be reconstituted and dispensed with the label “clinical trial” by the pharmacy of each site which is authorized to carry out preparations made necessary for biomedical research including experimental drugs. In the event of it being impossible for the PUI to prepare the treatment under study (too many demands for clinical study preparations, less staff on weekends and bank holidays), the latter will dispense the product labeled “clinical trial” to a third-party unit not involved in the PTH trial. The verum treatment **labeled “clinical trial”**, will be prepared according to the SmPC of the product (acyclovir). The list of “third-party” units is provided in section 20.1 of this protocol. A specific prescription is provided for when a “third-party” unit is called for.

Length of treatment: treatment shall be administered for 14 days

Placebos

Placebos are prepared in exactly the same way as ganciclovir (CMV arm of the study) and as acyclovir (HSV arm of the study), thus requiring a powder to be reconstituted. A pharmaceutical processing company authorized by the Agence Nationale de la Sécurité du Médicament (French National Agency for Drug Safety) will be in charge of manufacturing, packaging and labeling these placebos. Given that the study is double-blind, dosage, methods of administration and length of treatment are identical to those of the respective verum.

As of February 14, 2016, the placebo for ganciclovir will be reconstituted and dispensed with the label "clinical trial" by the pharmacy of each site which is authorized to carry out preparations made necessary for biomedical research including experimental drugs. In the event of it being impossible for the PUI to prepare the treatment under study (too many demands for clinical study preparations, less staff on weekends and bank holidays), the latter will dispense the product labeled "clinical trial" to a third-party unit not involved in the PTH trial The placebo will be made up of a pouch of 5% glucose solution (50 or 100 ml) **labeled "clinical trial"**. The list of "third-party" units is given in section 20.1 of this protocol. A specific prescription is provided for when a "third-party" unit is called for.

As of November 16, 2016, the placebo for acyclovir will be reconstituted and dispensed with the label "clinical trial" by the pharmacy of each site which is authorized to carry out preparations made necessary for biomedical research including experimental drugs. In the event of it being impossible for the PUI to prepare the treatment under study (too many demands for clinical study preparations, less staff on weekends and bank holidays), the latter will dispense the product labeled "clinical trial" to a third-party unit not involved in the PTH trial The placebo will be made up of a pouch of 5% glucose solution (50 or 100 ml) **labeled "clinical trial"**. The list of "third-party" units is given in section 20.1 of this protocol. A specific prescription is provided for when a "third-party" unit is called for.

Packaging, labeling and storage conditions

The trial products (verum and placebos) will be packaged double-blind by the same processing pharmaceutical company in charge of making the placebo. For the economical and ergonomic management of the drugs, they will be presented in packs of 7 days treatment. A total of 1000 kits for 7 days will be made (2 kits per patient for the full 14 days of treatment) (500 of verum, 500 of placebo). The fact that there is a higher number of kits than the number of patients included can be explained by the number of kits "immobilized "in each center.

The products' labeling contents shall comply with French Health Regulation requirements (art R. 5123 of the Code de Santé Publique) and shall include the following information:

- Name, address and telephone number of the Sponsor
- Trial reference (PTH-CMV or PTH-HSV) to identify the biomedical research study
- The product's pharmaceutical form, route of administration and its identification and dosage
- Manufacturing batch number
- Date of expiry
- Indications necessary for its correct storage
- The following statement "For biomedical research, use under strict medical surveillance".
- Treatment kit number
- Space for the patient's ID

Each kit of treatment will have a label. This label will include, among other things, the treatment kit number. When the kit is given to the investigator, this number will be copied in the eCRF.

Label model

ASSISTANCE PUBLIQUE - HOPITAUX DE MARSEILLE
80 rue Brochier 13005 Marseille 05 tel 04 91 38 27 47
PHRC 2011 – étude PTH (EudraCt : 2012-003312-30)
Investigator coordinator : Pr PAPAZIAN

Powder for perfsuion use (intravenous)
Ganciclovir 500mg or Placebo

BATCH: …………… Expiry on: …………
Manufacturing code :……………….
Treatment kit number………………….
Patient initials: …………………..

Keep out of childrens' reach and sight. Respect the prescribed dose.
Use under strict medical surveillance (art R5123 of CSP)
Store below 30°C
DRUG FOR CLINICAL TRIAL ONLY

ASSISTANCE PUBLIQUE - HOPITAUX DE MARSEILLE
80 rue Brochier 13005 Marseille 05 tel 04 91 38 27 47
PHRC 2011 – étude PTH (EudraCt : 2012-003312-30)
Investigator coordinator : Pr PAPAZIAN

Powder for perfusion use (intravenous)
Acyclovir 500mg or Placebo

BATCH: …………… Expiry on: …………

Manufacturing code :……………….
Treatment kit number:…………………
Patient initials: …………………..
Keep out of childrens' reach and sight. Respect the prescribed dose.
Use under strict medical surveillance (art R5123 of CSP)
Store below 25°C
DRUG FOR CLINICAL TRIAL ONLY

As of February 14, 2016, with regards to the CMV study subset, reconstituted ganciclovir or its placebo will be issued by the pharmacy of each site. The type of product resulting from randomization (verum or placebo) as well as the randomization number will be given to the on-site pharmacy. Ganciclovir will be prepared according to recommendations given in the Vidal: inject 10 ml water for injectable preparation into the bottle of ganciclovir - shake thoroughly – remove the required quantity and dilute it in a pouch or flask of either 50 or 100 ml of 5% glucose solution. Reconstituted mix can be stored for 24h maximum in the fridge. The placebo will be prepared using a pouch or flask of 50 or 100 ml 5% glucose solution with nothing added. Each site will define their own common strategy for the preparation of verum or placebo (type of solvent: 5% glucose solution, flask or pouch of 50 or 100 ml). A label will be stuck on each reconstituted product including:

- Name, address and telephone number of the Sponsor
- Trial reference (PTH-CMV) to identify the biomedical research study
- Indications necessary for its correct storage
- The following statement "For biomedical research, use under strict medical surveillance".
- Space for the patient's reference number
- Space for the patient's ID

ASSISTANCE PUBLIQUE - HOPITAUX DE MARSEILLE
80 rue Brochier 13005 Marseille 05 tel 04 91 38 27 47
PHRC 2011 – PTH-CMV trial (EudraCt : 2012-003312-30)
Investigator coordinator : Pr PAPAZIAN
Injectable solution of Ganciclovir or Placebo … mg/mL
Ganciclovir or placebo …………mg / Glucose 5% ………….. mL
IV Infusion
Randomization number : …………………………Prescription N°:………………
Date/time of preparation : …………………………………………………………….
Date/time of expiry: ……………………………………….. (stability : 24 hours)
Patient's Last Name, First name :………………………………………………………………
Keep out of childrens' reach and sight. Respect the prescribed dose.
Use under strict medical surveillance (art R5123 of CSP)
Keep refrigerated (between + 2 and + 8 °C) – Do not refreeze
DRUG FOR CLINICAL TRIAL ONLY

As of November 16, 2016, with regards to the HSV study subset, reconstituted aciclovir or its placebo will be issued by the pharmacy of each site. The type of product resulting from

randomization (verum or placebo) as well as the randomization number will be given to the on-site pharmacy. Acyclovir will be prepared according to recommendations given in the Vidal: inject 20 ml water for injectable preparation into the bottle of 500 mg acyclovir – shake thoroughly – remove the required quantity and dilute it in a 100 ml pouch or flask of saline solution. The reconstituted mix can be stored at room temperature (temperature < 25°C) for 12 hours maximum. Placebo will be prepared using a pouch or flask containing 100 ml of saline solution with nothing added. A label will be stuck on each reconstituted product including:

- Name, address and telephone number of the Sponsor
- Trial reference (PTH-HSV) to identify the biomedical research study
- Indications necessary for its correct storage
- The following statement “For biomedical research, use under strict medical surveillance”.
- Space for the patient’s reference number
- Space for the patient’s ID

ASSISTANCE PUBLIQUE - HOPITAUX DE MARSEILLE
80 rue Brochier 13005 Marseille 05 tel 04 91 38 27 47
PHRC 2011 – PTH-HSV trial (EudraCt : 2012-003312-30)
Investigator coordinator : Pr PAPAZIAN
Injectable solution of Acyclovir or Placebo … mg/mL
Acyclovir or placebo …………mg / Saline solution ………….. mL
IV infusion
Randomization number : …………………………Prescription N°:………………
Date/time of preparation : ……………………………………………………………..
Date/time of expiry: …………………………………………… (stability : 12 hours)
Patient’s Last Name, First name :………………………………………………………………
Keep out of childrens’ reach and sight. Respect the prescribed dose.
Use under strict medical surveillance (art R5123 of CSP)
Store at room temperature (<+25 °C)
DRUG FOR CLINICAL TRIAL ONLY

3. Distribution

Distribution and stock management of treatments under study in the different pharmacies for internal use will be performed by the Hôpitaux Sud pharmacy of the AP-HM in Marseille. To enable economical stock management, treatments will be distributed to the different centers in several phases depending on available stock.

As of February 14, 2016, each investigation site will reconstitute ganciclovir or issue the placebo under an identical form, for the CMV study subset. The investigator-clinician will draw up a prescription and transmit it to the pharmacy. This will include the identity of the

patient, their inclusion number, the words PTH-CMV trial, that day's serum creatinine results, the dosage prescribed.

As of November 16, 2016, each investigation site will reconstitute acyclovir or issue the placebo under an identical form, for the HSV study subset. The investigator-clinician will draw up a prescription and transmit it to the pharmacy. This will include the identity of the patient, their inclusion number, the words PTH-HSV trial, that day's serum creatinine results, the dosage prescribed.

Early treatment

Treating early with antiviral drugs is a potentially important factor in limiting morbidity/mortality associated with *Herpesviridae*. The introduction of the verum (ganciclovir or acyclovir) or of the placebo must not be later than 24h after PCR results are known.

Responsibility concerning the treatment under study

The pharmacist is responsible for checking the products received by signing an acknowledgment of receipt for every product delivery. All of the products must be stored in locked premises with access strictly limited to staff appointed for the trial. Products will be stored according to the Sponsor's specifications, following the manufacturer's instructions and in compliance with applicable regulations.

The pharmacist is responsible for maintaining and keeping an accurate register of the quantities of product dispensed, used and returned for each individual. All of the treatments under study must be dispensed according to the investigator's prescription.

Any quality issue observed upon receipt or when using the treatment under study (defect of the state, aspect, documentation, labeling, date of expiry, etc….of the treatments under study) must be immediately reported to the sponsor, who will initiate a complaints process. In the event of the identification of a potential quality defect of the treatments under study, the sponsor can decide to initiate a recall process. In this case, the investigator or the pharmacist will be responsible for immediately treating the sponsor's demand, in order to recall the experimental drug and to eliminate any potential risks.

The Investigator may not in any case, provide the treatment under study to a third party, nor enable the treatment under study to be used outside the clinical trial protocol, nor dispose of the drug under study by any other means.

Criteria for stopping the treatment under study

- Treatment with ganciclovir or its placebo will be stopped in the event of:
  - Leukopenia < 1000/mm^3
  - Neutropenia ≤ 500/mm^3
  - Thromboctyopenia < 25000/mm^3
- Treatment with acyclovir or its placebo will be stopped in the event of acute kidney injury occurring under treatment and **considered by the physician investigator to be related to the study treatment** and defined by one of the following criteria from the AKIN-stage 3 classification (Mehta et al. Crit Care 2007): increase of serum creatinine levels by at least 3-fold compared to the level at randomization OR serum creatinine levels above or equal to 354 µmol/L OR oliguria (< 0.3 ml/kg/h in 24h or 12h of anuria) OR requiring renal replacement therapy.

4. Unblinding

Unblinding is the exceptional procedure consisting of revealing the treatment code for one of the patients. This is usually done once the trial has finished. Nevertheless, some specific circumstances require unblinding while the trial is going on: mainly in the event of an adverse effect for patient safety and to be able to set up appropriate care for this event by having all the facts. Unblinding before the end of the trial will only apply to the patient concerned. It will be obtained via the eCRF.

Compliance

Treatment compliance will be monitored by the nurse in charge of each patient, he/she will fill in an administration sheet, keep the empty vials and any potentially non-allocated vials.

A register accounting for products returned one by one will be held by the pharmacist at the investigation sites. The study monitor will periodically check the quantity of products held by the investigator or by the pharmacist, to check that the products used are being correctly managed.

At the end of the study, the CRA in charge of quality control will count the number of therapeutic units used. This data will be used to calculate observance and to identify deviations from the protocol.

Treatment returns and destruction

At the end of the trial, all of the non-used or partially used products and all of the packaging will be returned to the PUI at the hôpital de la Timone of the AP-HM.

Destruction will be performed there under the pharmacist's responsibility with the sponsor's permission.

6. Evaluation criteria

Main judgment criterion: number of days alive and weaned off MV defined at D60

The main objective of either arm is to show a significant reduction in morbi-mortality thanks to a preemptive strategy against *Herpesviridae*, shown by the difference (in favor of the group treated with ganciclovir in the CMV arm and by acyclovir in the HSV arm) in the number of days for which the patients are alive and weaned off MV, evaluated at 60 days after inclusion. To count, the weaning period must exceed 48h without invasive MV. Patients who deceased prior to D60 have, by definition, zero days alive and ventilator-free even if they were weaned off it for several days (David A. Schoenfeld, PhD; Gordon R. Bernard, MD; for the ARDS Network. Statistical evaluation of ventilator-free days as an efficacy measure in clinical trials of treatments for acute respiratory distress syndrome. Crit Care Med 2002; 30:1772–1777).

Some examples:

- Patient weaned at D10 post-inclusion: VFD D60 = 60 – 10 = 50
- Patient weaned at D70 post-inclusion: VFD D60 = 0
- Patient weaned from D12 to D20 but deceased on D40: VFD D60 = 0
- Patient weaned at D45 but deceased on D80: VFD D60 = 60 – 45 = 15.

In cases where the patient has several episodes of at least 48h ventilator-free, VFD is equal to the sum of ventilator-free days.

The delay of 60 days **post-inclusion** (frequently used in intensive care studies) enables to account for the fact that the impact on prognosis could lead to an increase in the number and the severity of bacterial infections subsequent to virus-induced immunosuppression. After D60, other intercurrent factors can make the results difficult to interpret.

Secondary judgment criteria

- Mortality at D60, in intensive care and in hospital

- Total length of MV for the survivors
- Total length of stay in ICU and in hospital
- Sequential organ failure assessed using the SOFA score on D3, D5, D7, D14, D21 and D28 post-inclusion
- Disappearance of oropharyngeal HSV replication and CMV PCR blood results becoming negative
- Occurrence of HSV bronchopneumonitis or active CMV infection (definitions chapter 7.9)
- Occurrence of bacteremia (positive blood culture results except for *Staphylococcus epidermidis*: 2 blood cultures).
- The occurrence of ventilator-associated pneumonia (VAP) will be looked into. Diagnosis will fulfill the following criteria:
    - 1) **Certain VAP**: at least one of the three following criteria:
        - positive pleural fluid culture
        - presence of a lung abscess on scan results
        - histological infectious pneumonia (foyer of consolidation with an accumulation of neutrophils in bronchioles and in the neighboring alveolar areas with or without tissue necrosis) from biopsy or autopsy
    - 2) **Probable VAP**: BAL or distal protected catheter (DPC) or tracheal aspiration with at least one bacteria present ($\geq 10^4$ cfu/ml for BAL - $\geq 10^3$ cfu/ml for DPC - $\geq 10^5$ cfu/ml for TA)
    - 3) **Possible VAP**: none of the above criteria despite altered CPIS ≥ 5 (see annex 2).
- At the end of the trial, an infectious disease assessment committee (2 experts) will be in charge of blindly validating the randomization arm, infectious events (sites, microorganisms, relapse) from the hospitalization report and from the eCRF.

ARDS occurrence will be noted. ARDS will meet the recently defined criteria *(The Berlin definition. The ARDS Definition Task Force JAMA. 2012;307(23):2526-2533 Published online May 21, 2012. doi:10.1001/jama.2012.5669).*

	Acute Respiratory Distress Syndrome
Timing	Within 1 week of a known clinical insult or new or worsening respiratory symptoms
Chest imaging[a]	Bilateral opacities—not fully explained by effusions, lobar/lung collapse, or nodules
Origin of edema	Respiratory failure not fully explained by cardiac failure or fluid overload<br>Need objective assessment (eg, echocardiography) to exclude hydrostatic edema if no risk factor present
Oxygenation[b]	
Mild	200 mm Hg $<$ PaO_2/FIO_2 $\leq$ 300 mm Hg with PEEP or CPAP $\geq$5 cm H_2O[c]
Moderate	100 mm Hg $<$ PaO_2/FIO_2 $\leq$ 200 mm Hg with PEEP $\geq$5 cm H_2O
Severe	PaO_2/FIO_2 $\leq$ 100 mm Hg with PEEP $\geq$5 cm H_2O

Abbreviations: CPAP, continuous positive airway pressure; FIO_2, fraction of inspired oxygen; PaO_2, partial pressure of arterial oxygen; PEEP, positive end-expiratory pressure.
[a] Chest radiograph or computed tomography scan.
[b] If altitude is higher than 1000 m, the correction factor should be calculated as follows: [PaO_2/FIO_2 × (barometric pressure/760)].
[c] This may be delivered noninvasively in the mild acute respiratory distress syndrome group.

- Septic shock occurrence will be defined by an association of sepsis combined with systolic arterial pressure dropping below 90 mm Hg (and/or mean arterial pressure below 60 mm Hg and/or systolic arterial pressure dropping by more than 40 mm Hg below baseline) despite sufficient volume expansion in the absence of any other cause of high blood pressure, requiring the use of vasopressors *(Members of the American College of Chest Physicians / Society of Critical Care Medicine. American College of Chest Physicians / Society of Critical Care Medicine Consensus Conference: Definitions for sepsis and organ failure and guidelines for the use of innovative therapies in sepsis. Crit Care Med 1992; 20:864-74).*

5. Evaluation of tolerance

The occurrence of side effects linked to acyclovir or to ganciclovir shall be investigated. Blood count results will be noted (for the CMV arm: every other day) and serum creatinine levels on D3, D5, D7 and D14. This will not be specific monitoring since it is usual in intensive care.

6. Other parameters to be noted

- **ICU admission**: age; gender; origin; reason for admission; severity of the underlying disease assessed using the McCabe & Jackson score; Simplified Acute Physiology Score (SAPS) II score; Sequential Organ Failure Assessment (SOFA) score; previous history of cancer, diabetes, alcoholism, chronic obstructive pulmonary disease, liver cirrhosis, chronic kidney failure (creatinine clearance < 60 $mL.min^{-1}$ or serum creatinine > 150

µmol.L^{-1} or chronic hemodialysis), recent blood transfusion (< 1 month), recent steroid treatment (less than a month before).

- **The day of inclusion**: SOFA, ventilator parameters, prior transfusions, Glasgow coma scale score, temperature, blood gas tests, vasopressors, antibiotics, steroids, dialysis, CPIS. Clinical Pulmonary Infection Score (CPIS) will also be assessed on D3, D7 and D14. We will be using a modified simplified version (Annex 2 : *Luna C. Resolution of ventilator-associated pneumonia: Prospective evaluation of the clinical pulmonary infection score as an early clinical predictor of outcome. Crit Care Med 2003; 31:676 –682*).
- **During monitoring**:
    - Use of steroids and/or statins

7. Study course

Selection

- Length of screening phase: 30 days
- PCR twice per week.

Inclusion

- As soon as PCR results are positive
- Verification of inclusion and exclusion criteria
- Information and collection of the informed consent form for the interventional trial

Randomization

We will be using an electronic randomization process using internet. A telephone hotline open 24/7 will be operational for any issue related to the protocol. We shall use randomization by minimization, stratified by site, length of MV upon inclusion and number of existing organ failures upon inclusion. During randomization, the presence of inclusion criteria and the absence of exclusion criteria will be checked.

Monitoring from D1 to D14 after inclusion

Calculation of SAPS 2 and SOFA scores on the day of inclusion. SOFA assessment is repeated on D3, D5, D7 and D14 (more or less 24h). Vital status and MV status. Occurrence of complications related to the treatment under study. Occurrence of ventilator-associated

pneumonia, bacteremia, ARDS, septic shock, HSV bronchopneumonitis, active CMV infection.

Monitoring from D14 to D60

SOFA score on D21 and D28. Vital status and MV status. Occurrence of ventilator-associated pneumonia, bacteremia, ARDS, septic shock, HSV pneumonia, active CMV infection.

Virology monitoring from D1 to D30

PCR will be performed twice per week, the same frequency as during screening. The results will no longer be transmitted to the clinicians. HSV and CMV PCR will be stopped when leaving intensive care or on D30 post-inclusion at the latest.

Visit on D60

Vital stats and MV status.

Summary of patient monitoring

	**Screening phase D4-D30**	**Inclusion**	**D 3**	**D 5**	**D 7**	**D 14**	**D 21**	**D 28**	**End* treatment**	**D60**
**Inclusion criteria**	X	X								
**SOFA/CPIS**		X	X	X	X	X	X	X	X	
**Blood count/ serum creatinine**		X	X		X	X			X	
**Pregnancy test**		X								
**PCR**	X	Twice per week until leaving ICU or D30 maximum								
**Vital status and weaning**									X	X

* If treatment discontinued before D14

Treatment of declared HSV or CMV infection

If, following inclusion, there is an onset of HSV bronchopneumonitis and/or active CMV infection, curative therapy may be prescribed by the clinician in charge of the patient. If this

infection occurs during the 14 days with the treatment under study and the investigator considers that it requires treating, the treatment under study will be stopped.

Active CMV infection is defined by antigenemia > 10 cells/200 000 cells and/or positive qualitative PCR on BAL and/or IgM positive WITH associated clinical or biological symptoms (transaminases > 3 times the norm) (digestive = positive biopsy).

HSV bronchopneumonitis is defined according to the 3 following criteria: clinical suspicion of ventilator-associated pneumonia, HSV detected in distal lung secretions (collected by BAL, DPC…) and a cytopathic effect on cells collected during lung aspiration.

Early withdrawal from the trial

The conditions for early withdrawal from the trial are as follows:

- withdrawal of consent
- adverse event related to one of the products under study (see chapter 12.5)
- occurrence of active CMV infection and/or HSV pneumonia (see chapter 7.9)

In the event of early withdrawal from the trial, a full final assessment must be carried out for each patient and the patients must have an end of trial visit within 30 days of stopping the treatment.

Adverse Effects occurring within 30 days from stopping treatment in patients who withdrew from the trial early must be documented, once the investigator has been informed.

8. Data collection and monitoring

Case report form

Data concerning patients included in the trial will be grouped in an eCRF filled in by the investigators.

The eCRF will be set up via a partnership with the network Recherche En Ventilation Artificielle (REVA) who has developed a standard eCRF adapted to intensive care that is already used in several PHRC including OVNI.

Each eCRF includes the center letter (one letter), the inclusion number (4 digits), the first three letters of the patient's last name, the first two letters of the patient's first name and the date of inclusion.

Role of CRA coordinators

Two CRA coordinators will be placed under the responsibility of the principal investigators. One CRA coordinator (0.33 FTE) is linked to the La Pitié site (Paris). One CRA coordinator (0.66 FTE) is linked to the Hôpital Nord site (Marseille).

They will be assigned the following tasks:

- distribution of the treatment to the different centers and treatment stock management controlled by Dr Penot-Ragon, Pharmacie des Hôpitaux Sud in Marseille (CRA Marseille)
- setting up the e-CRF in collaboration with the principal investigators and the REVA network (CRA Paris)
- monitoring inclusions – reminders to investigators in order to stimulate inclusions (CRA Marseille and CRA Paris)
- sending monthly information to investigators in newsletters (CRA Marseille)
- remote checking of eCRF data entry (CRA Marseille and CRA Paris)
- following-up queries (CRA Marseille and CRA Paris)
- organizing periodic meetings with the sponsor (CRA Marseille and CRA Paris)
- monitoring the budget along with the sponsor (CRA Marseille)

Quality control

Quality control will be performed on all of the case report forms in the study (100% of data) by the sponsor.

9. Data management

Data management during the trial

A protocol for database transfer will be set up before the beginning of data collection. This will describe the database structure and function: tables, variables, labels, codes and formats used, reference data, checks carried out during data entry, as well as the means of transfer. A transfer test will be carried out in order to validate the correct transmission of all of the data.

Data coding

Concomitant treatments will be coded according to ATC classification. Medical history, concomitant illnesses and adverse events will be coded using the MedDRA dictionary.

Data validation

Electronic consistency checks will be performed according to a data management plan which will be drawn up for the protocol. If inconsistencies are detected, correction requests (DCF) will be sent to the investigators and inconsistency removal will be continued until all of the corrections have been entered and validated.

Review of data validation

A meeting to review blind data will be held for each study arm (HSV/CMV).

Freezing the database

The database will be frozen once all of the corrections requested from the investigators and the decisions made during the data review meetings have been integrated into the data files. The database will be frozen according to CIC-CPCET procedures with audit trails, and documented by a declaration of database freeze. The database will be archived before statistical analyses begin. Any further modification will be subject to an unfreeze request.

Data storage

The trial database as well as any change history (audit trails) will be saved and held by the CIC-CPCET for the legal archiving period. The CIC-CPCET will ensure archiving of the frozen database in SAS format.

10. Statistical method

Planned number of patients and justification

We hypothesize that a 14-day treatment with acyclovir immediately after positive HSV PCR results would enable to increase the number of days alive and ventilator-free by 8 days compared to the placebo group. According to data in literature, the variability of the number of days patients stay alive and ventilator-free corresponding to HSV inclusion criteria has a standard deviation of 20 days (2). Concerning the CMV arm, we based it on the same hypotheses: a standard deviation of 20 days and an expected difference of 8 days between both groups (ganciclovir vs placebo).

Considering these hypotheses, for a bilateral study and with an expected power defined at 80%, the number of individuals to include in each group is 97 patients. However, this number of individuals needs to be adjusted by the asymptotic relative efficiency factor for

the Mann-Whitney test (non-Gaussian distribution) (Hollander M, Wolfe D. Nonparametric statistical methods, 2nd edition. New York NY, John Wiley and Sons, Inc, 1999). The correction factor applied is 0.864. The number of individuals per group is therefore 112 (97 / 0.864). In order to be sure of having 112 exploitable observations per group and to account for any secondary attrition, 120 patients will be included in each treatment group.

A total of 240 patients will be included in the CMV arm (120 will receive ganciclovir, 120 will receive the placebo) and 240 patients will be included in the HSV arm (120 will receive acyclovir, 120 will receive the placebo).

Twenty percent of screened patients could have positive CMV PCR results (Jaber et al. Chest 2005, Chiche et al. Crit Care Med 2009, Limaye et al. JAMA 2008). We have applied the same hypothesis for HSV (keeping in mind that there is a higher percentage of HSV). Finally, approximately 40% of screened patients would therefore have positive PCR results for CMV or HSV. **Therefore a total of at least 1200 patients will need to be screened.**

Randomization method

Randomization will be performed independently for each arm of the study (CMV and HSV) following the same procedure.

Within each arm, included patients will be randomly allocated by draw to either treatment group: placebo or verum (ganciclovir or acyclovir). Randomization will be stratified according to the center, the length of MV prior to inclusion and the presence of organ failure. The length of MV prior to inclusion is split into 2 levels: between 4 and 14 days, and between 15 and 30 days. This stratification aims to limit the risk of non-comparability between groups, given that the incidence of positive CMV and HSV results is linked to the length of ventilation. Organ failure is also split into 2 levels: < 2 or ≥ 2 organ failures according to the SOFA score.

Minimization will be used to attribute patients into a group. Randomization will be electronic. A telephone hotline will be available 24/7.

Statistical analysis

Statistical analysis will start after the database has been frozen, the report from the data review meeting has been approved, and the Statistical Analysis Plan (SAP) has also been approved. The methods given in this section may be updated during the study, justified and finalized in the SAP.

Analysis of joint trial data (screening phase) and of the CMV arm will be carried out by the CIC-UPCET (Elisabeth Jouve, Hopital de la Timone, AP-HM) in collaboration with the SSPIM Sud (Vanessa Pauly, Pr Xavier Thirion, Hôpital Sainte Marguerite, AP-HM). Data analysis of the HSV arm will be carried out by Pr Alain Mallet (URC Pitié-Salpêtrière, AP-HP).

Withdrawal from the trial and deviations from the protocol

Patient status will include the number of selected patients, the number of patients included in each arm, then for each study arm: the number of patients given the treatment under study, the number of patients per visit, the number of patients who withdrew from the trial with the reasons for stopping.

Major protocol deviations will be described in detail. The patients for whom there is a protocol deviation will be identified and classified. Only the cases of patients with major protocol deviations will be discussed in the clinical study report. Major deviations will be used to define the per protocol population. In this hypothesis, analysis of the intention-to-treat population prevails over analysis of the per protocol population which will be complementary.

Populations analyzed

In each study arm, the following populations will be analyzed:

- For tolerance: all of the patients treated (T), *i.e.* all of the patients who received at least one dose of the treatment under study and for whom at least one measurement of tolerance data is available.
- For efficiency:
    - Full Analysis Set (FAS), all of the patients randomized except patients who withdrew their consent. This is the “intention-to-treat” population.
    - Per Protocol (PP), *i.e.* patients from the tolerance population who participated in the whole trial without any major deviation.

General statistical approach

Descriptive statistics will be presented per treatment group and per visit, for measured values with absolute variations (differences) and/or relative variations (%) in comparison with inclusion. Quantitative variables will be given as mean, standard deviation, median, quartiles

Q1 (25th percentile) and Q3 (75th percentile), minimum and maximum. Qualitative variables will be given as relative frequency and percentage.

Reference values (baseline) are defined as being the last measurement available prior to randomization.

Missing data will not be replaced.

Statistical tests used to compare both of the treatment groups will be defined according to the type of variable and to respect conditions for the validity of tests: Student's t-test (or Mann-Whitney test), ANOVA (or Kruskall-Wallis test), chi-squared test (or Fisher's exact test).

The normality of the variables will be assessed graphically and with the Shapiro-Wilk test. Mathematical transformations may be applied in order to normalize distribution data if necessary.

No sub-group analysis is planned in the protocol to analyze efficiency. We will privilege use of multivariate analysis which accounts for adjustment factors if it seems necessary to take concomitant parameters into account (potential bias): models for analysis of variance or of covariance for quantitative parameters, logistic regression model for qualitative parameters.

No intermediate statistical analysis is planned.

The threshold for significance is set at $p < 0.05$.

Analysis will be carried out using SAS software.

Description of patients and comparability between groups

This analysis will be carried out independently for each study arm: CMV and HSV.

The populations studied will be described according to the main parameters measured upon inclusion.

All of the parameters collected during the screening phase and the day PCR results became positive will be described per studied population and per treatment group.

Comparability between treatment groups will be checked with the following parameters:

- socio-demographic data and context of admission in intensive care (age, gender, reason for admission...)
- severity of patients (SOFA, SAPS II scores, McCabe & Jackson classification), medical history and comorbidity, treatment taken during the previous month and concomitant on D1)
- ventilation parameters (length, ..)
- other physiological parameters and scores (Glasgow coma scale, CPIS)

This analysis will identify any potential adjustment factors.

Efficiency analysis

This analysis will be carried out independently for each study arm: CMV and HSV.

10.8.1. Primary analysis

The main judgment criterion is the number of days alive and weaned off MV on D60 right-censored: *ventilator-free days and alive* (VFD). (definition in 6.1)

The number of VFD will be given on D60 and compared between groups using a method of means comparison which will be chosen according to:

- whether adjustment is necessary or not, special attention will be paid to the state of severity of patients on randomization;
- the distribution of this variable, supposedly not normally distributed, and therefore the possible use of a normalizing transformation.

According to the case, the strategy for analysis will be the following:

- normal distribution (or possible normalization), no adjustment: Student's t- test
- normal distribution (or possible normalization), adjustment: ANOVA-ANCOVA

- non normal distribution, no adjustment: Mann-Whitney test
- non normal distribution with adjustment: ANOVA-ANCOVA confirmed by Mann-Whitney test after stratification for the adjusted parameter.

10.8.2. Secondary analyses

All of the secondary judgment criteria will be given per treatment group. Parameters measured at several monitoring points will be given for each point with their variation compared to inclusion values.

The following will be compared between both treatment groups, using the Chi2 test (or Fisher's exact test):

- Mortality on D60
- Mortality in ICU
- Mortality in hospital
- Incidence of HSV bronchopneumonitis
- Incidence of active CMV infection- Reactivation rate
- Incidence of bacterial infections (ventilation-associated pneumonia, bacteremia) according to the classification: certain, probable, possible VAP (see paragraph 6.2)
- PCR results becoming negative
- Disappearance of oropharyngeal HSV reactivation
- Occurrence of severe or moderate ARDS
- Occurrence of septic shock

The following will be compared between both treatment groups using the Student's t-test (or Mann-Whitney test):

- Length of invasive MV
- Length of hospital stay in ICU
- Length of stay in hospital
- Number of days with organ failure (defined using the SOFA score) between inclusion and D28

10.8.3. Tolerance analysis

Clinical treatment tolerance is evaluated by analyzing adverse events and biological data.

10.8.4. Analysis of adverse events

All adverse events will be given per "organ system" and per "preferred term" (MedDRA) in summary tables. Adverse event frequency (%) will be calculated for the total number of events ($\%N_{EI}$) and for the number of patients ($\%N_P$).

Criteria in terms of adverse events are the number of patients who presented:

- at least one adverse effect,
- one adverse event for each identified preferred term,
- one adverse event for each identified organ system.

These criteria apply to any adverse event whatever its relationship with the product under study. They will be analyzed for the tolerance population, presented as a whole and by treatment group.

Serious adverse events (whether during treatment or not, and whatever their supposed causal relationship with the products under study) will be listed and summarized.

Adverse events leading to a halt by administration will be listed and summarized for each individual, whether they took place during treatment or not, and whatever their supposed causal relationship with the product under study.

10.8.5. Analysis of biological data

Blood count and serum creatinine levels measured on D3, D7 and D14 will be summarized per treatment group as values and variation from baseline.

Altered renal function will be described at each assessment by:

- the number of patients per level of serum creatinine in the CMV arm.
- the number of patients per level of serum creatinine in the HSV arm.

(see paragraph 5.5 criteria for modifying or stopping the treatment under study).

11. Treatments

7. Treatment under study

Observance for products under study will be provided as a percentage: number of days treated / number of days of trial.

8. Concomitant treatment

Concomitant treatment throughout the trial will be given according to its ATC code.

12. Study calendar

Study length

The inclusion phase will last for 30 months (progressive opening and closing of the 14 centers depending on the sponsor's availability). The trial will last for 36 months.

Length of participation of each person

Each patient will participate for 60 days.

Research calendar

**Date**	**Action**
July 2012	Application to the Comité de Protection des Personnes (French Independent Ethics Committee) and to the ANSM (French National Agency for Drug Safety)
September 2012	Manufacture of the placebo and product packaging
November 2012	Pilot Committee meeting:<br>- Define the different phases of the study<br>- Distribute documents to the centers<br>- Transport active products and placebos to the centers
January 2014	Start of inclusion
May 2016	End of inclusion
July 2016	End of patient follow-up
September-November 2016	Quality control and data entry
December 2016	Data analysis
January 2017	Pilot Committee meeting:<br>- Present and validate results<br>- Organize data valorization (congresses and scientific papers)

13. Adverse event management

9. General Definition

A Serious Adverse Event (SAE) is any event that:

- Results in death,
- Is life-threatening,
- Causes prolongation of existing hospitalization,
- Results in medically significant persistent disability or serious temporary incapacity.

The terms *disability and incapacity* correspond to any temporary or permanent physical or mental disability, clinically significant and having an effect on the patient's physical activity and/or quality of life.

The term *medically significant* is any clinical event or laboratory result that the investigator considers to be serious and does not correspond to the severity criteria defined above. It may put the patient at risk and require a medical intervention to avoid one of the outcomes corresponding to the severity criteria given above.

10. Definition of an expected serious adverse event

An E-SAE is one of the events already mentioned in the summary of characteristics of the product under study which already has a marketing authorization.

11. Definition of an unexpected serious adverse event

A U-SAE is an event that is not mentioned in or differs from the summary of characteristics of the product under study by its type, intensity, or progression.

12. Intensity criteria

Intensity criteria must not be confused with severity criteria which serve as a guide to define report obligations.

The intensity of events will be estimated using the NCI-CTC grading scale version 3.0 (toxicity graded from 1 to 5). The intensity of adverse events not listed in this scale will be graded according to the following qualifiers:

- **Light (grade 1):** does not affect the patient's usual activities of daily living
- **Moderate (grade 2):** perturbs the patient's usual activities of daily living

- **Severe (grade 3):** prevents the patient's usual activities of daily living
- **Very severe (grade 4):** life-threatening
- **Death (grade 5)**

13. Conduct to be followed in the case of a serious adverse event

The investigator informs pharmacovigilance at the DRC of the AP-HM about all of the **Unexpected Serious Adverse Events (U-SAE) as well as the Expected Serious Adverse Events (E-SAE) particularly including: death, leukopenia < 1000/mm^3, thrombocytopenia < 25000/mm^3, acute kidney injury considered by the physician investigator to be related to the treatment under study and defined as serum creatinine levels three times higher than on randomization OR serum creatinine above or equal to 354 µmol/L OR oliguria (< 0.3 ml/kg/h in 24h or 12h of anuria) OR requiring renal replacement therapy,** whether attributed or not to the research study and which occur during the trial or within 30 days of treatment being administered. **The treatment on trial will be stopped in the event of one of these SAE.**

Reports will be made by sending the *"serious adverse event report"* form (inserted in the patient's clinical report form) filled in as accurately as possible, dated and signed, within **24 working hours** of the observation, by fax to

Direction de la Recherche Clinique (DRC) de l'AP-HM

Pharmacovigilance- Cellule Contrôle - Qualité

Tel. : +33(0)4 91 38 28 94 – Fax : +33(0)4 91 38 14 79

For each event the investigator will note:

- Its description as clearly as possible using medical terminology,
- Whether the event was expected or unexpected,
- Its intensity,
- The start and end dates of the event,
- Measures taken and whether corrective treatment was required or not,
- If the treatment on trial was discontinued,
- Its progression. In the case of a non fatal event, progression must be studied until healing or returning back to previous state or stabilization of any potential sequelae,
- The causal relationship between this event and the treatment on trial or a constraint related to the research study (period without treatment, supplementary tests required for research etc.),

- The causal relationship with the drug(s) on trial, the illness being treated, another illness or another treatment.

The investigator must also enclose the following with the SAE report form, whenever it is possible:

- A copy of the hospitalization or prolongation of hospitalization report,
- A copy of the autopsy report,
- A copy of all the supplementary exam results carried out, including pertinent negative results, providing laboratory reference values,
- Any other document that he/she considers useful and pertinent.

All of these documents must be anonymized.

Extra information may be requested (by fax, by phone or during a visit) **from the investigator by pharmacovigilance.** Within **48 hours** the latter must send the sponsor the ***"supplementary information for an unexpected SAE"*** form (inserted in the patient observation form) correctly filled in.

14. SAE follow-up

The investigator is responsible for adapted medical follow-up of patients until the event is resolved or stabilized or until the patient dies. **Sometimes this can imply that follow-up will be pursued after the patient has left the trial.**

He/she transmits supplementary information to Pharmacovigilance (PV) of the DRC at the AP-HM using the SAE report form (ticking the box Follow-up n° X to show that it is a follow-up and not a primary report) within <u>48 hours</u> of receiving it; He/she also transmits the final follow-up when the SAE is resolved or stabilized.

He/she keeps all of the documents concerning the adverse effect in order to, if necessary, complete previously transmitted information. He/she answers requests made by PV of the DRC in order to document the primary observation.

14. Surveillance committee

Role

Its role will be to check that the trial is run correctly, especially the incidence of adverse events. It will be regularly consulted as serious adverse events are reported.

Composition

The surveillance committee will be made up of two independent university-hospital intensive care physicians not taking part in the trial (Pr Eric Maury – Hôpital Saint-Antoine, Paris, Pr Elie Azoulay – Hôpital Saint-Louis, Paris) and one methodologist (Pr Christian Mélot – Hôpital Erasme de Bruxelles).

15. Legal and ethical aspects

Sponsor's role

This is defined by law 2004-806 of 9 August 2004. For this research study, the Assistance Publique – Hôpitaux de Marseille will be the sponsor and the Direction Régionale de la Recherche Clinique which covers its regulatory assignments will play a decision-making role. The APHM will take out an insurance policy for the course of this trial. Finally, this work will be performed in collaboration with the Clinical Investigation Center of the Hôpital de la Timone.

This project's sponsor is the Assistance Publique des Hôpitaux de Marseille. The Sponsor will keep regulatory watch over it. It will submit the project to the responsible authorities for their approval.

This project is within the frame of an interventional biological research study, according to article L.1121-1, relating to a product mentioned in article L.5311-1 of the Code de la Santé Publique (French Health Regulations) (drug); it comes under the new regulatory measure which applies to research "organized and practiced on human beings with the aim to develop knowledge in biology and medicine" which is the Loi de Santé Publique (French Public Health Law) n°2004-806 of 9 August 2004 relating to the public health policy and its implementing decrees of 27 August 2006, aiming to bring French regulations in line with European law. For this reason it will be submitted to the Comité de Protection des Personnes Sud-Méditerranée V (South Mediterranean Ethical Committee) and to the Agence Nationale de Sécurité du Médicament et des produits de santé (ANSM; French National Agency for Drug Safety) for their favorable opinion. An information sheet will be given to patients and informed consent must be obtained. It will be written in compliance with regulatory recommendations, notably providing the study objective, the advantages and the risks linked to the trial, the trial course and all of the legal provisions to which patients are entitled.

This research study will be performed in compliance with good clinical practice, representing a group of quality requirements in ethical and scientific fields, which must be respected during planning, implementation, conduct, follow-up, quality control, auditing, data collection, analysis and expression of the results. Respecting this good clinical practice will guarantee the protection of rights, protection and safety of the individuals taking part in this research study and preservation of their anonymity as well as the credibility (integrity, authenticity, verifiability) and accuracy of data and results in this research study.

Quality control and quality assurance

Each patient will be given a unique identification number, which will be generated on randomization and transmitted to the physician in charge of inclusion. This number will be copied in the eCRF. Quality control will be performed on-site by a clinical research associate.

This study will be conducted following the AP-HM standardized procedures when our Institute is the research sponsor. The course of research on all of the investigation sites as well as patient care will be performed in compliance with Helsinki recommendations and with Good Clinical Practice.

The sponsor's representatives will visit the investigation centers according to the defined procedure. Thus, a set-up meeting will be held in the presence of the sponsor at the opening of each center. The same thing will take place for the closure of each center. Monitoring visits will take place for every 5 inclusions or every semester at the least if there have been less than 5 inclusions.

Submission to the ethics committee

This study will be submitted to the Comité de Protection des Personnes Sud-Méditerranée V (South Mediterranean Ethics Committee) following the sponsor's approval and obtaining insurance cover. This committee's opinion will be reported in the form sent to the appropriate authority by the sponsor before the research study commences.

Data processing – Confidentiality

Patients and/or their families will be individually informed in writing about the creation of a computer file for the management of collected data. People will be informed that they have the possibility to apply to the person in charge of the study for a right of access or modification of data concerning them.

Clinical and biological data will be stored in a database, the access to which will be controlled by an individual identification system for users (passwords). Computer hardware on which the data is stored will not be linked to a transmission network.

Concerning data processing for this project, which has a research objective in the health sector, it is within the framework of legal requirements, especially the law of 9 August 2004, and will only include data which does not allow direct or indirect identification of the individuals concerned. It will be carried out in compliance with baseline methodology certified by the Commission nationale de l'informatique et des libertés (CNIL; French National commission for the protection of personal data and individual liberties) and set up in conjunction with the advisory committee for data processing in matters of research in the health sector, drawn up with the aim of simplifying formalities (Decision of 5 January 2006. Baseline methodology MR-001).

Medical and non-medical staff involved in this research are sworn to medical and occupational secrecy with relation to the data concerning patients gathered during the study. Information gathered from patients will remain strictly confidential. It will be kept in paper format inside locked premises. It will be entered into a computer and automatically processed. This computer processing will not allow direct or indirect identification of the patients. The entire data may only be consulted by the principal investigator and the sponsor's representatives, or be transmitted to the Authorized Health Authorities if necessary. The study will be declared to the CNIL in compliance with applicable legislation (Loi Informatiques et Libertés (French law on Data Protection) of 6 January 1978, amended by the law of 1 July 1994 and the Decree of 9 May 1995). The patient (and his/her representatives) who participates must be informed about the type of data being processed, its finality, the identity of individual and legal entities who will be sent this data. He/she retains the right to access and rectify this data via the physician of his/her choice, as well as the right to object. In accordance with the law of 4 March 2002 relating to patients' rights and the quality of the health system, the global results of the trial can be transmitted to the patients and their representatives at their request, directly or via the physician of their choice.

Criteria for stopping the trial early

The sponsor and the investigator are entitled to stop the trial at any time before all of the planned subjects are included, for valid scientific, safety or administrative reasons. In this event, both parties agree to procedures based on individual evaluation per patient after critical analysis.

The trial can be discontinued and/or be analyzed before all of the subjects are included in the following cases:

- If the trial is not led in accordance with the procedures planned in the protocol such as it was approved by administrative authorities.
- In the event of a suspected modification of the benefit-risk ratio (management of the number of adverse events)
- At the sponsor's discretion if he/she thinks it necessary; furthermore the sponsor is entitled to request the exclusion of a subject in the event of a (major) breach of protocol, for administrative reasons, or for any other valid or ethical reason.

16. Publication rules

The first, fourth, one before last and last places of both of the two main articles (one for the CMV arm, one for the HSV arm) will be reserved for the principal investigators. The second place will be assigned to the center which included the most patients in that given arm. The third, fifth, sixth, seventh and eighth places will be assigned to the five centers which included the most patients (after the first center).

17. Cost and additional costs of research

The sponsors ensures trial organization and covers the supply of elements such as the protocol, investigation site files, experimental drugs…Potential additional costs named in article R.2038 of the Code de la Santé Publique (French Public Health Regulations) will be subject to an agreement negotiated between the AP-HM and the representative of the investigation sites, taking into account the financial means available to the sponsor within the frame of promotion activities.

Two **CRA coordinator**/logistic assistance places (0.33 FTE and 0.66 FTE) will be credited over 30 months to work in close collaboration with the principal investigators of the 2 coordinator sites: Paris (HSV arm) and Marseille (CMV arm). They will be en liaison with the different centers participating in the study during the trial.

In order to help each clinical team to gather data, a flat rate of 200 euros will be granted **per patient included in the interventional study**. This will enable the sponsor's quality control CRA to limit his/her monitoring visits.

In order to reimburse the virology departments involved in the study, a flat rate of 150 euros will be granted **for each patient screened**.

18. Responsibilities of the sponsor / Insurance

In compliance with the law Huriet, the sponsor will take out insurance to cover its civil liability in the event of harmful consequences of research for the person taking part in it (art. L.1121-7),

19. References

1. Chiche L, Forel JM, Roch A, Guervilly C, Pauly V, Allardet-Servent J, Gainnier M, Zandotti C, Papazian L. Active cytomegalovirus infection is common in mechanically ventilated medical intensive care unit patients. *Crit Care Med* 2009;37:1850-1857.
2. Luyt CE, Combes A, Deback C, Aubriot-Lorton MH, Nieszkowska A, Trouillet JL, Capron F, Agut H, Gibert C, Chastre J. Herpes simplex virus lung infection in patients undergoing prolonged mechanical ventilation. *Am J Respir Crit Care Med* 2007;175:935-942.
3. Osawa R, Singh N. Cytomegalovirus infection in critically ill patients: A systematic review. *Crit Care* 2009;13:R68.
4. Vincent A, La Scola B, Forel JM, Pauly V, Raoult D, Papazian L. Clinical significance of a positive serology for mimivirus in patients presenting a suspicion of ventilator-associated pneumonia. *Crit Care Med* 2009;37:111-118.
5. Muenzer JT, Davis CG, Chang K, Schmidt RE, Dunne WM, Coopersmith CM, Hotchkiss RS. Characterization and modulation of the immunosuppressive phase of sepsis. *Infect Immun* 2010;78:1582-1592.
6. Gandhi MK, Khanna R. Human cytomegalovirus: Clinical aspects, immune regulation, and emerging treatments. *Lancet Infect Dis* 2004;4:725-738.
7. Cook CH, Zhang Y, Sedmak DD, Martin LC, Jewell S, Ferguson RM. Pulmonary cytomegalovirus reactivation causes pathology in immunocompetent mice. *Crit Care Med* 2006;34:842-849.
8. Heininger A, Jahn G, Engel C, Notheisen T, Unertl K, Hamprecht K. Human cytomegalovirus infections in nonimmunosuppressed critically ill patients. *Crit Care Med* 2001;29:541-547.
9. Jaber S, Chanques G, Borry J, Souche B, Verdier R, Perrigault PF, Eledjam JJ. Cytomegalovirus infection in critically ill patients: Associated factors and consequences. *Chest* 2005;127:233-241.
10. Limaye AP, Kirby KA, Rubenfeld GD, Leisenring WM, Bulger EM, Neff MJ, Gibran NS, Huang ML, Santo Hayes TK, Corey L, et al. Cytomegalovirus reactivation in critically ill immunocompetent patients. *Jama* 2008;300:413-422.
11. Papazian L, Fraisse A, Garbe L, Zandotti C, Thomas P, Saux P, Pierrin G, Gouin F. Cytomegalovirus. An unexpected cause of ventilator-associated pneumonia. *Anesthesiology* 1996;84:280-287.
12. Cook CH, Martin LC, Yenchar JK, Lahm MC, McGuinness B, Davies EA, Ferguson RM. Occult herpes family viral infections are endemic in critically ill surgical patients. *Crit Care Med* 2003;31:1923-1929.
13. Heininger A, Vogel U, Aepinus C, Hamprecht K. Disseminated fatal human cytomegalovirus disease after severe trauma. *Crit Care Med* 2000;28:563-566.
14. Papazian L, Doddoli C, Chetaille B, Gernez Y, Thirion X, Roch A, Donati Y, Bonnety M, Zandotti C, Thomas P. A contributive result of open-lung biopsy improves survival in acute respiratory distress syndrome patients. *Crit Care Med* 2007;35:755-762.
15. Bruynseels P, Jorens PG, Demey HE, Goossens H, Pattyn SR, Elseviers MM, Weyler J, Bossaert LL, Mentens Y, Ieven M. Herpes simplex virus in the respiratory tract of critical care patients: A prospective study. *Lancet* 2003;362:1536-1541.
16. Porteous C, Bradley JA, Hamilton DN, Ledingham IM, Clements GB, Robinson CG. Herpes simplex virus reactivation in surgical patients. *Crit Care Med* 1984;12:626-628.

17. Ong GM, Lowry K, Mahajan S, Wyatt DE, Simpson C, O'Neill HJ, McCaughey C, Coyle PV. Herpes simplex type 1 shedding is associated with reduced hospital survival in patients receiving assisted ventilation in a tertiary referral intensive care unit. *J Med Virol* 2004;72:121-125.
18. De Vos N, Van Hoovels L, Vankeerberghen A, Van Vaerenbergh K, Boel A, Demeyer I, Creemers L, De Beenhouwer H. Monitoring of herpes simplex virus in the lower respiratory tract of critically ill patients using real-time pcr: A prospective study. *Clin Microbiol Infect* 2009;15:358-363.
19. Francois-Dufresne A, Garbino J, Ricou B, Wunderli W. Ards caused by herpes simplex virus pneumonia in a patient with crohn's disease: A case report. *Intensive Care Med* 1997;23:345-347.
20. Tuxen DV, Cade JF, McDonald MI, Buchanan MR, Clark RJ, Pain MC. Herpes simplex virus from the lower respiratory tract in adult respiratory distress syndrome. *Am Rev Respir Dis* 1982;126:416-419.
21. Arata K, Sakata R, Iguro Y, Toda R, Watanabe S, Eitsuru Y. Herpes simplex viral pneumonia after coronary artery bypass grafting. *Jpn J Thorac Cardiovasc Surg* 2003;51:158-159.
22. Byers RJ, Hasleton PS, Quigley A, Dennett C, Klapper PE, Cleator GM, Faragher EB. Pulmonary herpes simplex in burns patients. *Eur Respir J* 1996;9:2313-2317.
23. Linssen CF, Jacobs JA, Stelma FF, van Mook WN, Terporten P, Vink C, Drent M, Bruggeman CA, Smismans A. Herpes simplex virus load in bronchoalveolar lavage fluid is related to poor outcome in critically ill patients. *Intensive Care Med* 2008;34:2202-2209.
24. Tuxen DV, Wilson JW, Cade JF. Prevention of lower respiratory herpes simplex virus infection with acyclovir in patients with the adult respiratory distress syndrome. *Am Rev Respir Dis* 1987;136:402-405.

20. Annexes

15. List of the centers having accepted to participate

Institute	Site	Intensive care unit	Third party unit
APHM	Hôpital Nord	Pr PAPAZIAN	Polyvalent intensive care unit
APHM	Hôpital Nord	Pr LEONE	DRIS intensive care unit
APHM	Hôpital de la Timone	Pr GAINNIER	Continuous monitoring unit
Marseille	Hôpital Européen	Dr ALLARDET-SERVENT	Continuous monitoring unit
APHP	Hôpital Pitié-Salpêtrière	Pr CHASTRE – Dr LUYT	Continuous monitoring unit
APHP	Hôpital Pitié-Salpêtrière	Dr Q. LU – Pr ROUBY	Continuous monitoring unit
APHP	Hôpital Pitié-Salpêtrière	Pr DUGUET – Pr SIMILOWSKI	Continuous monitoring unit
APHP	Hôpital Cochin	Pr MIRA	Not concerned
APHP	Hôpital Bichat	Pr WOLFF – Pr TIMSIT	Continuous monitoring unit
APHP	Hôpital Georges Pompidou	Pr FAGON – Pr DIEHL	Not concerned
Nîmes CHU	Hôpital Caremeau	Pr LEFRANT	Continuous monitoring unit
Clermont-Ferrand CHU	Hôpital Estaing	Pr CONSTANTIN	Continuous monitoring unit
Clermont-Ferrand CHU	Hôpital Montpied	Pr SOUWEINE	Continuous monitoring unit
HCL	Hôpital Edouard Herriot	Dr RIMMELE	Continuous monitoring unit
HCL	Hôpital de la Croix-Rousse	Pr GUERIN	Not concerned
Versailles CH	Hôpital Mignot	Dr BEDOS	Not concerned
Montpellier CHU	Hôpital Saint-Eloi	Pr JABER	Continuous monitoring unit
	Hôpital Lapeyronie	Dr JUNG	Continuous monitoring unit
Limoges CHU	Hôpital Dupuytren	Dr CLAVEL	Continuous monitoring unit
Grenoble CHU	Hôpital Nord	Pr SCHWEBEL	Continuous monitoring unit
Nantes CHU	Intensive care medicine and Resuscitation	Dr LASCARROU	Continuous monitoring unit
	Surgical intensive care	Pr ASEHNOUNE	Continuous monitoring unit
Orléans CH	Polyvalent intensive care	Dr BOULAIN	Continuous monitoring unit

16. Clinical pulmonary infection score (CPIS)

Modified CPIS (after *Luna C. Resolution of ventilator-associated pneumonia: Prospective evaluation of the clinical pulmonary infection score as an early clinical predictor of outcome Crit Care Med 2003; 31:676 –682*)

**Temperature, °C**	≥ 36.5 et ≤ 38.4	**0**
	≥ 38.5 et ≤ 38.9	**1**
	≥ 39.0 ou ≤ 36.0	**2**
**Leukocytes, G/l**	≥ 4 and ≤ 11	**0**
	< 4 or > 11	**1**
**Quantity of tracheal secretions**	Little	**0**
	Average	**1**
	Abundant	**2**
**Aspect of tracheal secretions**	Purulent	**+ 1**
**PaO_2/FiO_2**	> 240 or ARDS	**0**
	≤ 240 and no ARDS	**2**
**Frontal chest imaging**	No infiltrate	**0**
	Diffuse and/or heterogeneous infiltrates	**1**
	Localized infiltrate	**2**

Total modified CPIS : 0 to 10

Initial statistical analysis plan (finalized on September 14, 2018, by Mamadou Hassimiou Diallo)

This SAP concerns only outcome criteria until day 60. The analysis of the randomized controlled-trial PTH (Preemptive Treatment for Herpesviridae) will be performed by including all randomized patients that gave their consent for use of their data (or whom next of kin gave his/her consent when follow-up consent was not possible).

Primary outcome

The primary outcome is the number of ventilator-free days at day 60, i.e. days alive without mechanical ventilation (VFD).

Secondary outcomes

The following outcomes will be evaluated at day 60 following randomization:

- Mortality
- Intensive care unit (ICU) mortality
- Hospital mortality
- Incidence of herpes simplex virus (HSV) bronchopneumonitis
- Incidence of cytomegalovirus active infections
- Incidence of bacterial infections (ventilator-associated pneumonia, bacteremia)
- Negativation of oropharyngeal HSV PCR
- Occurrence of acute respiratory distress syndrome
- Occurrence of septic shock
- Kinetics of organs failure assessed by the Sequential Organ failure Assessment (SOFA) score at days 3, 5, 7, 14, 21 and 28
- Duration of mechanical ventilation
- ICU length of stay
- Hospital length of stay

Tolerance

Tolerance of the study drug will be evaluated by analysis of adverse events, cause of study drug withdrawal and biological data (mainly creatinine). Study drug observance will be evaluated by the number of effective treatment days / number of theoretical treatment days.

Methodology of statistical analysis

Descriptive statistics will be presented by treatment group (i.e., acyclovir or placebo) and by visit. For measured variables, absolute change (difference) and relative change (percentage) will be expressed as a change from inclusion. Quantitative variables will be described using mean, standard deviation, median, quartiles (25th and 75th), minimum and maximum values. Qualitative variables will be described using frequency (number and percentage).

Baseline values are defined as the first available measure before randomization. Missing data will not be replaced.

Statistical tests to compare the 2 groups will be determined according to variables and compliance with test application conditions: Student test (or Mann-Withney test), ANOVA (or Kruskall-Wallis), khi-two (or Fischer's exact test).
Variables normality will be graphically assessed using the Shapiro-Wilks test. Mathematical transformation could be realized to normalize data if necessary.

No subgroup analysis is prespecified by the protocol for efficacy analysis. The use of multivariable analysis, to take into account confounding factors will be used if it appears necessary (in case of obvious bias): variance analysis or covariance analysis for quantitative parameters, logistic regression for qualitative parameters.
Level of significance is set at $p < 0.05$.

Analysis will be performed on an intention to treat basis. A per protocol analysis will be performed in all patients having participated to the study without major deviation, having received at least one dose of study drug, and for whom at least one data on tolerance is available.

Primary outcome is ventilator-free days at day 60. This outcome will be compared between groups using a test of comparison of means that will be determined according to

- The need for adjustment, with particular warning on the severity of patients at randomization
- The distribution of this variable, a priori not normally distributed, and thus the potential use of a normalizing transformation

According to the findings during the analysis, the strategy of analysis will be the following:

- Normally distributed variable (or possible normalization), no adjustment: Student test
- Normally distributed variable (or possible normalization), need for adjustment: ANOVA-ANCOVA
- Not normally distributed variable, no adjustment: Mann-Whitney test
- Not normally distributed variable, need for adjustment: ANOVA-ANCOVA confirmed by a Mann-Whitney test after stratification on the adjustment parameter.

All secondary outcomes will be described according to the treatment group. For the parameters that are measured at different time, they will be presented with their change as compared to inclusion.

The following parameters will be compared using the Chi2 test (or Fischer's exact test)
- Day 60 mortality
- ICU mortality
- Hospital mortality
- Incidence of HSV bronshopneumonitis
- Incidence of active CMV infection
- Incidence of bacterial infections (ventilator-associated pneumonia-bacteremia)
- Negativation of oropharyngeal HSV PCR
- Occurrence of acute respiratory distress syndrome
- Occurrence of septic shock

The following parameters will be compared using the Student test (or Mann-Whitney test) at day 60:

- Duration of invasive mechanical ventilation
- Duration of ICU length of stay
- Duration of hospital length of stay
- Duration of treatment with study drug

Adverse events, acute renal failure and reasons for stopping treatment will be described as numbers (percentage) for each treatment group. Study drug observance will be assessed by a percentage: number of effective treatment days / number of theoretical treatment days.

Analysis of biological data will be performed on blood count (white blood cells and platelets counts) and creatinine levels measured at day 3, 7 and 14 and will be resumed (mean, median, standard deviation, IQR, minimal and maximal values) according to treatment group, with raw value and change from baseline

Final statistical analysis plan (finalized on February 28, 2019, by Mamadou Hassimiou Diallo)

This SAP concerns only outcome criteria until day 60 and day 90 (on data prospectively collected). The analysis of the randomized controlled-trial PTH (Preemptive Treatment for Herpesviridae) will be performed by including all randomized patients that gave their consent for use of their data (or whom next of kin gave his/her consent when follow-up consent was not possible).

Primary outcome

The primary outcome is the number of ventilator-free days at day 60, i.e. days alive without mechanical ventilation (VFD).

Secondary outcomes

The following outcomes will be evaluated at day 60 following randomization:

- Mortality
- Intensive care unit (ICU) mortality
- Hospital mortality
- Incidence of herpes simplex virus (HSV) bronchopneumonitis
- Incidence of cytomegalovirus active infections
- Incidence of bacterial infections (ventilator-associated pneumonia, bacteremia)
- Occurrence of acute respiratory distress syndrome
- Occurrence of septic shock
- Kinetics of organs failure assessed by the Sequential Organ failure Assessment (SOFA) score at days 3, 5, 7, 14, 21 and 28
- Duration of mechanical ventilation
- ICU length of stay

Supplementary secondary outcomes (post hoc analyses):

- Description of the following parameters: temperature, white blood cells count, radiological score, modified clinical pulmonary infection score, bilirubin level, platelets count, Glasgow coma score
- Occurrence of renal replacement therapy
- Time between randomization and occurrence of ARDS at day 60
- Time between randomization and occurrence of septic shock at day 60
- Time between randomization and weaning from mechanical ventilation at day 60
- Survival between randomization and day 60
- Subgroup analysis (defined by par les caractéristiques de randomisation: duration of mechanical ventilation before randomization, number of organ failure at randomization). Subgroups analyses were performed on the following outcomes : ventilator-free days and day-60 mortality
- Number of ventilator-free days at day 90 (performed on data prospectivelly collected)
- Day 90 mortality and global survival between inclusion and day 90.

Tolerance

Tolerance of the study drug will be evaluated by analysis of adverse events, cause of study drug withdrawal and biological data (mainly creatinine). Study drug observance will be evaluated by the number of effective treatment days / number of theoretical treatment days.

Methodology of statistical analysis

Descriptive statistics will be presented by treatment group (i.e., acyclovir or placebo) and by visit. For measured variables, absolute change (difference) and relative change (percentage) will be expressed as a change from inclusion. Quantitative variables will be described using mean, standard deviation, median, quartiles (25th and 75th), minimum and maximum values. Qualitative variables will be described using frequency (number and percentage). Except otherwise specified, all analyses will be performed on an intention-to-treat basis. A per protocol analysis, excluding patients with protocol deviation (patients without inclusion criteria, or those treated with study drug for less than 7 days for other reasons that death) will be performed.

Baseline values are defined as the first available measure before randomization. Missing data

Primary outcome is ventilator-free days at day 60. This outcome will be compared between groups using Mann-Whitney test

All secondary outcomes will be described according to the treatment group. For the parameters that are measured at different time, they will be presented with their change as compared to inclusion.

The following parameters will be compared using the Chi2 test (or Fischer's exact test)
- Day 60 mortality
- ICU mortality
- Hospital mortality
- Incidence of HSV bronshopneumonitis
- Incidence of active CMV infection
- Incidence of bacterial infections (ventilator-associated pneumonia-bacteremia)
- Occurrence of acute respiratory distress syndrome
- Occurrence of septic shock

A risk difference (and its 95% confidence interval) will also be calculated to compare day-60 mortality between the 2 groups.

The following parameters will be compared using the Student test (or Mann-Whitney test) at day 60:
- Duration of invasive mechanical ventilation
- Duration of ICU length of stay
- Duration of hospital length of stay
- Duration of treatment with study drug

Adverse events, acute renal failure and reasons for stopping treatment will be described as numbers (percentage) for each treatment group. Occurrence of at least one adverse event will be compared between the 2 groups using a Chi2 test (or Fisher's exact test).
Study drug observance will be assessed by a percentage: number of effective treatment days / number of theoretical treatment days and compared using Student or Wilcoxon test.

Analysis of biological data will be performed on blood count (white blood cells and platelets counts) and creatinine levels measured at day 3, 7 and 14 and will be resumed (mean, and 95% CI according to treatment group.

Analysis of supplementary secondary outcomes (post hoc analyses):

Ventilatory and blood gazes parameters between inclusion and day 28 will be resumed using the mean (95% CI) value according to treatment group. Occurrence of acute renal failure will be expressed as number of patients between inclusion and day 285 in each group.

The following outcomes will be compared using Kaplan-Meier analysis and logrank test:
- Time between randomization and occurrence of ARDS at day 60
- Time between randomization and occurrence of septic shock at day 60
- Time between randomization and weaning from mechanical ventilation at day 60
- Survival between randomization and day 60
- Survival between inclusion and day 90

Ventilator-free days at day 90 will be compared between groups using Mann-Withney test. Mortality at day 90 between groups will be compared using Chi2 test.

Regarding the subgroups analysis
- We will first test the interaction between groups and stratification criteria (number of organ failure at randomization and duration of mechanical ventilation at randomization) using a linear regression for ventilator-free days and logistic regression for day-60 mortality
- A Wilcoxon test will be performed on ventilator-free days in each subgroup
- A calculation of risk difference (with its 95% CI) will be performed on day-60 mortality in each subgroup.

All analyses will be performed with a bilateral alpha risk of 5%.

Secondary outcome collected but not analyzed

Due to the high number of missing data on hospital length of stay (roughly 39% of data are missing), this criteria will not be analyzed

Secondary outcome not collected

For financial reason, follow-up oropharyngeal HSV PCR (to look for negativation of oropharyngeal HSV during treatment) was not performed. This criteria will therefore not be analyzed.

Summary of changes in the statistical analysis plan

Supplementary secondary outcomes:

- Description of the following parameters: temperature, white blood cells count, radiological score, modified clinical pulmonary infection score, bilirubin level, platelets count, Glasgow coma score
- Occurrence of renal replacement therapy
- Time between randomization and occurrence of ARDS at day 60
- Time between randomization and occurrence of septic shock at day 60
- Time between randomization and weaning from mechanical ventilation at day 60
- Survival between randomization and day 60
- Subgroup analysis (defined by par les caractéristiques de randomisation: duration of mechanical ventilation before randomization, number of organ failure at randomization). Subgroups analyses were performed on the following outcomes : ventilator-free days and day-60 mortality
- Number of ventilator-free days at day 90 (performed on data prospectivelly collected)
- Day 90 mortality and global survival between inclusion and day 90.

Secondary outcome collected but not analyzed because lot of missing data:

- Duration of hospital length of stay

Secondary outcome not collected:

- Negativation of oropharyngeal HSV PCR
