# Supplementary material for: Efficacy of Acyclovir to Suppress Herpes Simplex Virus Oropharyngeal Reactivation in Patients Who Are Mechanically Ventilated: An Ancillary Study of the Preemptive Treatment for Herpesviridae (PTH) Trial
Source: JAMA Netw Open. 2021 Dec 20;4(12):e2139825. doi: 10.1001/jamanetworkopen.2021.39825 (PMC8689380; doi:10.1001/jamanetworkopen.2021.39825)
Supplement: Supplement 2. — eMethods. eFigure. Study Flowchart [file jamanetwopen-e2139825-s002.pdf]

Supplemental Online Content

Luyt CE, Hajage D, Burrel S, et al. Efficacy of acyclovir to suppress herpes simplex virus oropharyngeal reactivation in patients who are mechanically ventilated: an ancillary study of the Preemptive Treatment for Herpesviridae (PTH) trial. *JAMA Netw Open*. 2021;4(12):e2139825. doi:10.1001/jamanetworkopen.2021.39825

eMethods.

eFigure. Study Flowchart

This supplemental material has been provided by the authors to give readers additional information about their work.

© 2021 Luyt CE et al. *JAMA Network Open.*

eMethods.

Patients included in 2 centers (Hôpital Pitié-Salpêtrière, Paris and Hôpital Nord, Marseille) had weekly oropharyngeal swabs during study follow-up. These swabs were collected at randomization, days 7, 14 and 21 post-randomization, with a tolerated delay of +/- 3 days for sampling. Swabs were either processed on a day to day basis (for patients included in Marseille), or frozen and further analyzed (for patients included in Paris). Herpes simplex virus was detected in these swabs using polymerase chain reaction using commercial kits on both sites (Roche Diagnostics for Marseille and Biomérieux for Paris). Patients included in the PTH trial in other centers had no follow-up swab and could therefore not be included in the present study.

Missing samples (n=16) were managed using the last observation carried forward as follow: in case of missing sample between 2 positive samples, the missing sample was considered as positive. In case of missing sample between 2 negative samples, the missing sample was considered as negative. If the missing sample followed a positive sample and preceded a negative one, the missing sample was considered as positive. No patient had consecutive missing samples.

Statistical analysis

Data are expressed as median [interquartile range (IQR)], mean (±SD) or mean (95% confidence interval [CI]), as appropriate. Between-group comparisons used Student's t-test or the Mann–Whitney U-tests for continuous variables according to the variable distribution, i.e., normal or not. For categorical variables, between-group comparisons used χ^2 or Fischer's exact tests.

Cumulative incidences of HSV shedding, extubation and death were calculated at randomization and days 7, 14 and 21 post-randomization and compared using a log-rank test.

© 2021 Luyt CE et al. *JAMA Network Open.*

Time to stop HSV shedding was evaluated using a competing risk analysis, taking into account extubation and death as competing events. Subdistribution hazard ratios (HR) with their 95% CI for HSV negative swab, extubation and death were obtained using Fine-Gray model.

All analyses were computed with R software, version 3.5.1 (R Project for Statistical Computing), at a 2-sided, 5% α level of significance.

© 2021 Luyt CE et al. *JAMA Network Open.*

eFigure. Study Flowchart

In grey, flow chart of the parent trial (PTH trial). In blue, patients included in the present ancillary study.

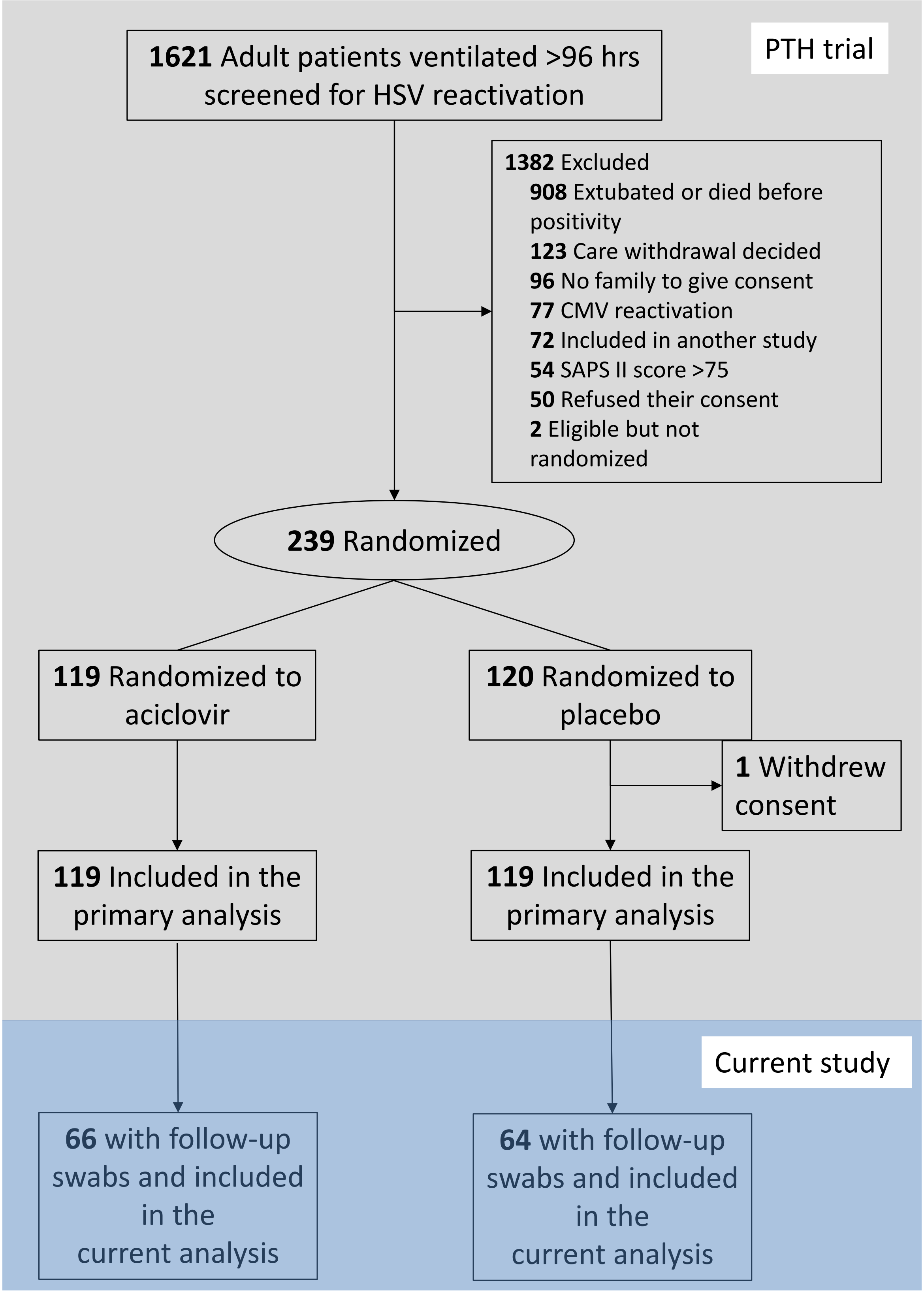

© 2021 Luyt CE et al. *JAMA Network Open.*
