# Supplementary material for: Efficacy of Acyclovir to Suppress Herpes Simplex Virus Oropharyngeal Reactivation in Patients Who Are Mechanically Ventilated: An Ancillary Study of the Preemptive Treatment for Herpesviridae (PTH) Trial
Source: JAMA Netw Open. 2021 Dec 20;4(12):e2139825. doi: 10.1001/jamanetworkopen.2021.39825 (PMC8689380; doi:10.1001/jamanetworkopen.2021.39825)
Supplement: Supplement 3. — Data Sharing Statement [file jamanetwopen-e2139825-s003.pdf]

Data Sharing Statement

Luyt CE, Hajage D, Burrel S, et al. Efficacy of acyclovir to suppress herpes simplex virus oropharyngeal reactivation in patients who are mechanically ventilated: an ancillary study of the Preemptive Treatment for Herpesviridae (PTH) trial. JAMA Netw Open. 2021;4(12):e2139825. doi:10.1001/jamanetworkopen.2021.39825

Data
Data available: Yes
Data types: Deidentified participant data
How to access data: on request sent to charles-edouard.luyt@aphp.fr
When available: With publication

Supporting Documents
Document types: Statistical/analytic code
How to access documents: on request sent to charles-edouard.luyt@aphp.fr
When available: With publication

Additional Information
Who can access the data: to researchers whose proposed used of the data have been approved by the principal investigator
Types of analyses: for any purpose
Mechanisms of data availability: After approval of a proposal, with a signed data agreement
